# A Selective SARS-CoV-2 Host-Directed Antiviral Targeting Stress Response to Reactive Oxygen Species

**DOI:** 10.1021/acscentsci.2c01243

**Published:** 2023-01-13

**Authors:** Cong Tang, Ana R. Coelho, Maria Rebelo, Hannah Kiely-Collins, Tânia Carvalho, Gonçalo J. L. Bernardes

**Affiliations:** † Instituto de Medicina Molecular João Lobo Antunes, Faculdade de Medicina, Universidade de Lisboa, Avenida Professor Egas Moniz, 1649-028, Lisboa, Portugal; ‡ Yusuf Hamied Department of Chemistry, University of Cambridge, Lensfield Road, Cambridge CB2 1EW, U.K.; § Champalimaud Foundation, Avenida de Brasília, 1400-038, Lisboa, Portugal

## Abstract

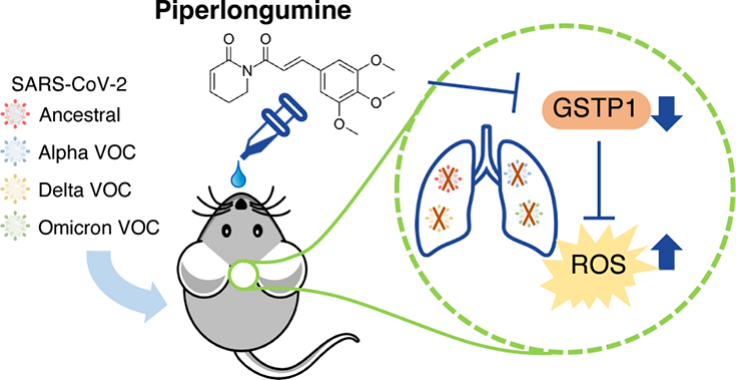

The emergence of severe acute respiratory syndrome coronavirus 2 (SARS-CoV-2) catalyzed the development of vaccines and antivirals. Clinically approved drugs against SARS-CoV-2 target the virus directly, which makes them susceptible to viral mutations, which in turn can attenuate their antiviral activity. Here we report a host-directed antiviral (HDA), piperlongumine (PL), which exhibits robust antiviral activity as a result of selective induction of reactive oxygen species in infected cells by GSTP1 inhibition. Using a transgenic K18-hACE2 mouse model, we benchmarked PL against plitidepsin, a HDA undergoing phase III clinical trials. We observed that intranasal administration of PL is superior in delaying disease progression and reducing lung inflammation. Importantly, we showed that PL is effective against several variants of concern (VOCs), making it an ideal pan-variant antiviral. PL may display a critical role as an intranasal treatment or prophylaxis against a range of viruses, expanding the arsenal of tools to fight future outbreaks.

## Abstract 2

Piperlongumine selectively induces reactive oxygen species in infected cells by targeting overexpressed GSTP1, thereby effectively protecting mice against severe acute respiratory syndrome coronavirus 2 (SARS-CoV-2) virus and variants of concern.

The severe acute respiratory syndrome coronavirus 2 (SARS-CoV-2) spread rapidly and unpredictably throughout the world when the first coronavirus disease 2019 (COVID-19) cases were discovered in Wuhan, Hubei Province of China.^1^ More than 616 million SARS-CoV-2 infections and 6.5 million fatalities have been documented as of September 28, 2022 (https://coronavirus.jhu.edu/map.html). Despite the discovery and dissemination of numerous safe and efficacious vaccines which contributed significantly to the control of the COVID-19 pandemic, the continuing emergence of new variants demonstrated that the virus is adapting to its new human host over time.^2^

So far, the discovery of antiviral compounds has mainly focused on direct-acting antivirals (DAAs), including the three drugs approved for clinical use, namely Remdesivir, PF-07321332 (paxlovid), and MK-4482/EIDD-2801 (molnupiravir).^3−5^ In addition, another class of antiviral compounds called host-directed antivirals (HDAs) or indirect-acting antivirals are believed to be more effective against SARS-CoV-2 variants of concern (VOCs) since host genes have a low propensity to mutate.^6^ However, their overall clinical superiority is still under investigation. Therefore, the discovery of novel host-directed antiviral compounds and identification of their underlying mechanism of action (MOA) are urgently needed.

Piperlongumine (Piplartine, (*E*)-1-(3-(3,4,5-trimethoxyphenyl)acryloyl)-5,6-dihydropyridin-2(1*H*)-one, PL) is an alkaloid/imide extracted from the long pepper (*Piper longum L.*). Long pepper is one of the most extensively utilized natural ingredients in Indian medical systems to cure various ailments.^7^ The antitumor activity of PL has been previously described,^8,9^ and in particular, its effect in cancer was characterized by the stress response to reactive oxygen species (ROS).^10^

In the present study, we discovered that PL exhibits potent anti-SARS-CoV-2 activity, with low micromolar potency *in vitro*, demonstrated in two different cell lines. By utilizing a transgenic mouse model of SARS-CoV-2 infection (K18-hACE2 mice), the antiviral effect of PL was evaluated *in vivo*. Plitidepsin (aplidin), an HDA compound, was included as a benchmark due to its highly potent antiviral activity. Our findings showed that PL is a more potent HDA relative to Plitidepsin, leading to a significant delay in body weight loss and reduced lung viral load.^11^ PL was administered intranasally, whereas Plitidepsin was administered subcutaneously; the intranasal route has been suggested to be preferable because the nasal mucosa is frequently the primary site of infection.^12^ More importantly, PL exerts robust antiviral activity against different viral variants *in vitro* and *in vivo*. The antiviral effect of PL is shown to be directed by the selective accumulation of ROS in infected cells. By inhibiting the upregulated pi-class glutathione *S*-transferase (GSTP1) in infected cells, PL induces the increase of ROS levels and decreases reduced glutathione (GSH). Subsequent ROS-mediated activation of mitochondrial antiviral-signaling protein (MAVS) triggers the downstream IFN-JAK-STAT pathway, which in turn leads to viral degradation. As an HDA, PL does not target the virus directly but rather affects the ROS level selectively in infected cells, suggesting that PL has the potential to become a pan-SARS-CoV-2 therapeutic and would be useful against emerging SARS-CoV-2 VOCs.

## Results

### PL is a Potent Inhibitor of SARS-CoV-2 Infection *in vitro*

**Figure 1:**
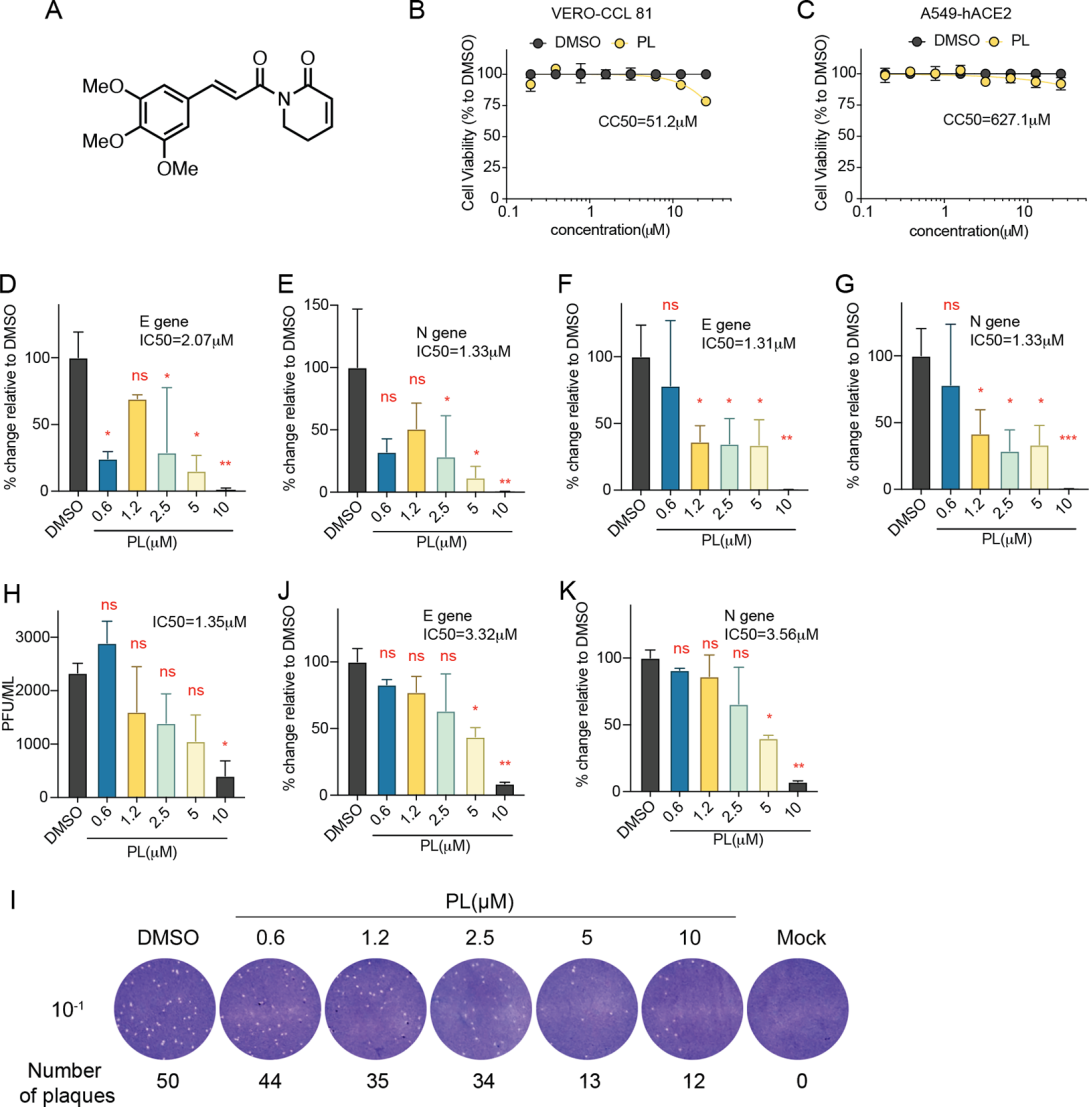
PL exhibits a robust antiviral activity against SARS-CoV-2 *in vitro*. (A) Chemical structure of piperlongumine (PL). Cytotoxicity of PL in VERO-CCL 81 (B) and A549-hACE2 (C) cell lines. Both cell lines were treated with indicated doses of PL, and cell viability was measured after 24 h. Dose–response antiviral activity of PL in VERO-CCL 81 cells where PL was added 1 h after infection, quantifying E gene (D) and N gene (E) of SARS-CoV-2 virus, evaluated by the qPCR assay. Dose–response antiviral activity of PL in VERO-CCL 81 cells with PL treatment 1 h prior to infection, quantifying E gene (F) and N gene (G) of SARS-CoV-2 virus, evaluated by the qPCR assay. (H) Plaque assay was performed in VERO-CCL 81 cells to determine the viral titers (amount of infectious virus) produced in cells pretreated with the indicated concentration of PL 1 h prior to infection. (I) Representative plaques from the plaque assay. The infection medium was diluted 10-fold and was indicated as 10^–1^. Mock was negative control incubated with medium. Dose–response antiviral activity of PL in A549-hACE2 cells which PL treated 1 h prior to infection, quantifying E gene (J) and N gene (K) of SARS-CoV-2 virus, evaluated by the qPCR assay. CC_50_ or IC_50_ values of PL were indicated under the curves. Data are presented as mean ± SD. One-way ANOVA test. *P < 0.05, **P < 0.01, ***P < 0.001.

To explore the effect of PL (Figure 1A) against SARS-CoV-2, we evaluated the antiviral activity of PL *in vitro* using two different cell lines. VERO-CCL 81 cells, isolated from *Cercopithecus aethiops* kidney, are widely used in microbiology due to their improved virus propagation potential, which leads to high-titer production viral stocks and rescue of clinical viral isolate.^13^ Human lung cancer cells A549 expressing angiotensin-converting enzyme 2 (HA-FLAG) (A549-hACE2) were also employed because the A549 cell line is extensively used for the study of respiratory infections, and the cell is susceptible to SARS-CoV-2 infection when transfected with the human ACE2 receptor. The cytotoxicity of PL was first examined in both cell lines, and results showed that both were tolerant to PL, with half-maximal cytotoxic concentration (CC_50_) values at 51.2 μM in VERO-CCL 81 cells (Figure 1B) and 627.1 μM in A549-hACE2 cells (Figure 1C), respectively. Considering this data, we decided to test the antiviral activity of PL within a range of 0.6 to 10 μM in both cell types. Specifically, cells were infected with the SARS-CoV-2 virus, PL was added 1 h after infection and then incubated for 24 h in VERO-CCL 81 cells. Measurement of viral load by PCR of the Envelop (E) gene and Nucleocapsid (N) gene of the SARS-CoV-2 virus showed that viral RNA decreased in a dose-dependent manner with 50% inhibitory concentration (IC_50_) of 2.07 μM for the E gene (Figure 1D) and 1.33 μM for the N gene (Figure 1E). In a prophylactic approach, PL was added to cells 1 h prior to infection and incubated for another 24 h after infection. We observed that PL inhibits SARS-CoV-2 replication, and viral RNA levels were also reduced in a dose-dependent manner with an IC_50_ of 1.31 μM for the E gene (Figure 1F) and 1.33 μM for the N gene (Figure 1G) in VERO-CCL 81 cells, suggesting that PL has the potential to be used in both prophylactic and therapeutic treatment settings. These observations were further supported by plaque assay results, which showed that PL could significantly reduce viral plaque forming units with an IC_50_ of 1.35 μM (Figure 1H–I). In A549-hACE2 cells, we observed the same inhibitory effect of PL, with IC_50_ of 3.32 μM for the E gene (Figure 1J) and 3.56 μM for the N gene (Figure 1K), as determined by qPCR. According to these results, PL is a robust inhibitor of SARS-CoV-2 infection with low (single-digit) micromolar potency.

### PL Shows *in Vivo* Antiviral Efficacy in a Mouse Model of SARS-CoV-2 Infection

**Figure 2:**
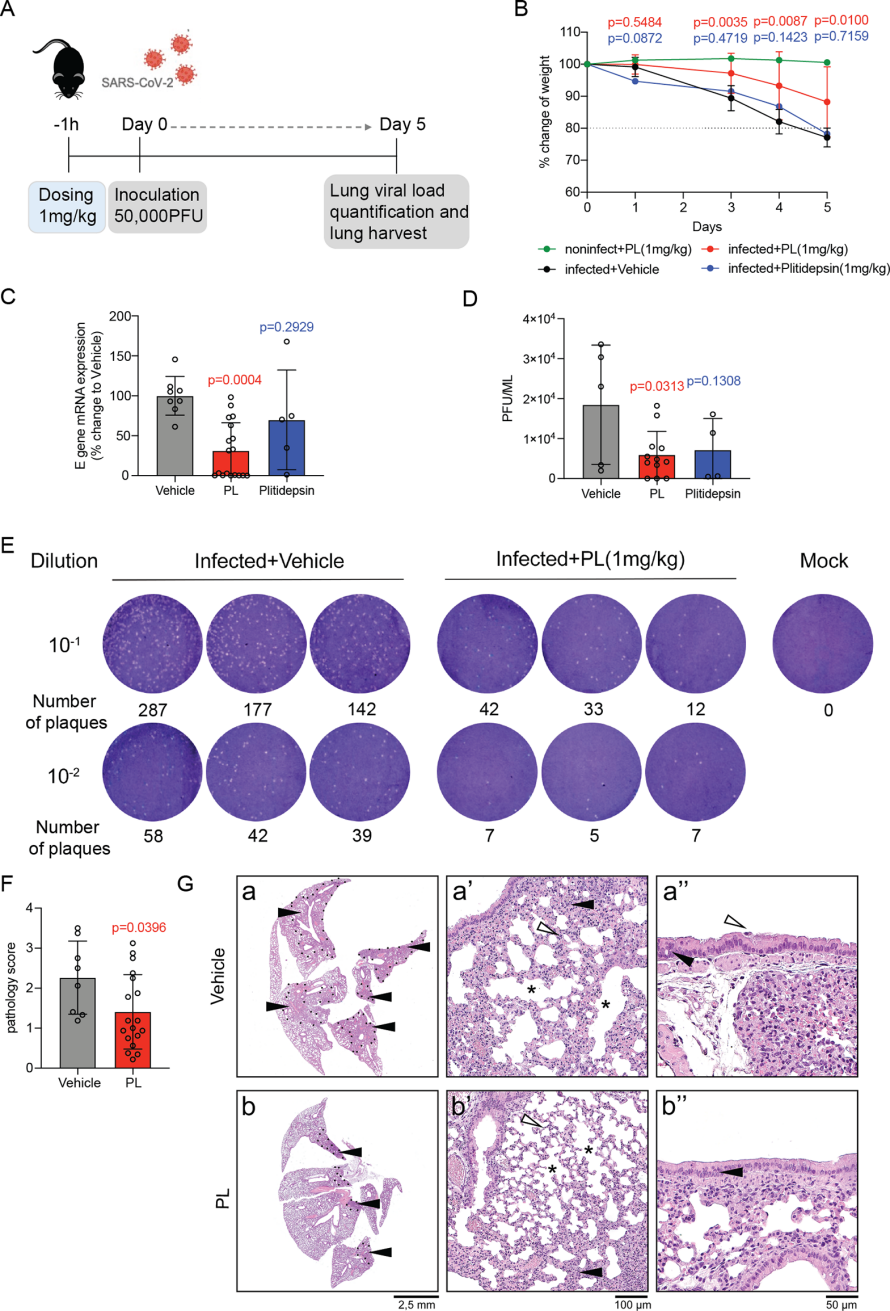
PL shows *in vivo* antiviral efficacy in the K18-hACE2 mouse model. (A) Schematic of the K18-hACE2 model of SARS-CoV-2 infection. 8–12 weeks-old K18-hACE2-transgenic mice were treated with 1 mg/kg PL (IN) (*n* = 18) or Plitidepsin (SC) (*n* = 6) or vehicle (*n* = 8) 1 h prior to intranasal inoculation of 5 × 10^4^ PFU of SARS-CoV-2 on Day 0. (B) Body weight change curve of vehicle control, PL- or Plitidepsin-treated infected mice, or PL-treated noninfected mice (*n* = 3). Mean ± SD. Multiple *t* test. Left lung viral titers of mice were determined by the qPCR assay (C) and plaque assay (D). Mean ± SD. One-way ANOVA test. (E) Representative images from the plaque assay. (F) Score of lung pathology in K18-hACE2 mice inoculated with SARS-CoV-2, untreated (vehicle), and treated with PL. Mean ± SD. Unpaired *t* test. (G) Representative microphotographs of the lung of K18-hACE2 mice inoculated with SARS-CoV-2 at Day 5 postinfection, untreated (a) and treated with PL (b). Depicted is the extent of the lesions in untreated/vehicle (a) and treated mice (b), which are in higher number (black arrowhead) and larger area (inside dashed lines) in untreated/vehicle animals. These lesions consisted in thickening of alveolar septae (white arrowhead), interstitial inflammation (black arrowhead), and mild emphysema (asterisk), seen at higher magnification (a′, b′), also with hyperplasia (black arrowhead) and necrosis of bronchiolar epithelium (white arrowhead) (a′′, b′′). Haematoxylin and eosin stain; original magnification 2.5×, (a, b), 20× (a′, b), and 40×′ (a′′, b′′).

After testing the efficacy of PL *in vitro*, we investigated whether PL would reduce morbidity and increase survival in a preclinical model of SARS-CoV-2 infection. We employed an established mouse model of SARS-CoV-2 infection, where transgenic K18-hACE2 mice expressing the human ACE2 receptor regulated by the keratin 18 promoter develop severe lung injury after SARS-CoV-2 inoculation.^14^ Extensive research into the preclinical anticancer potential of PL has resulted in a number of papers and patents (WO2009114126-A1, EP2276487-A1) for treating cancer.^8,9^ Results from these studies have revealed a well-established safety profile and pharmacokinetics. Here, dosing regimens and medication concentrations were selected based on the solubility of PL in previous anticancer and safety studies.^15^ We envisioned that an ideal route for administration of PL would be intranasal, because this would maximize airway and lung exposure and further activate protective immunity, hence preventing virus infection and transmission.^16^ Then, using noninfected mice, we determined that the drug was nontoxic via this route at 1 mg/kg, a dose for which we did not observe body weight loss or adverse effects, suggesting that 1 mg/kg of PL is a safe dose (Figure 2B). We also included a benchmark HDA drug in this *in vivo* study, Plitidepsin, as it has previously been reported to reduce viral loads in the lungs of mice by 2 orders of magnitude when administered prophylactically in mouse models of SARS-CoV-2 infection.^11^ Mice were administered with 1 mg/kg of PL or Plitidepsin 1 h prior to infection, inoculated with 5 × 10^4^ PFU/mouse of ancestral SARS-CoV-2 on Day 0, and monitored daily until Day 5 when the vehicle mice reached approximately 80% of initial body weight and showed severe signs of disease including hunched posture, respiratory distress, labored breathing, and decreased mobility. Mice were euthanized on Day 5, and lungs collected for viral load quantification or histopathology (Figure 2A). Control animals, noninfected but treated with PL 1 mg/kg, did not show weight loss or other clinical signs of disease (Figure 2B).

We observed that PL-treated infected mice showed a two-day delay in body weight loss and onset of symptoms compared to the vehicle group as well as the Plitidepsin-treated group. Of note, the vehicle group reached 90% of initial body weight on Day 3, while the PL-treated group reached the same level only on Day 5 (Figure 2B). Both qPCR and plaque assays were used for viral load quantification and showed that PL significantly reduced lung viral load (over 60% reduction assessed by the qPCR assay) on Day 5. This reduction is even more significant relative to a single dose of 1 mg/kg of Plitidepsin treatment (only 30% reduction) (Figure 2C–E). Histopathology of the lung (Figure 2F–G) showed reduced inflammation in PL-treated mice (pathology score of 1.40/4) in comparison to vehicle-treated mice (pathology score of 2.26/4) at Day 5, and decreased pulmonary edema, hyaline membranes formation, proliferation of bronchiolar epithelium, and hemorrhage in the PL-treated group. Heart, spleen, liver, kidney, brain, cerebellum, and nasal turbinates were also analyzed for disease-associated changes and evidence of toxicity of PL, and no lesions were observed in any of the organs (Figure S1). Altogether, these results showed that PL could inhibit the replication of SARS-CoV-2 and lung inflammation *in vivo*, providing compelling support for further clinical investigation of PL as a COVID-19 preventative drug.

### PL Inhibits SARS-CoV-2 VOCs Replication Both *in Vitro* and *in Vivo*

**Figure 3:**
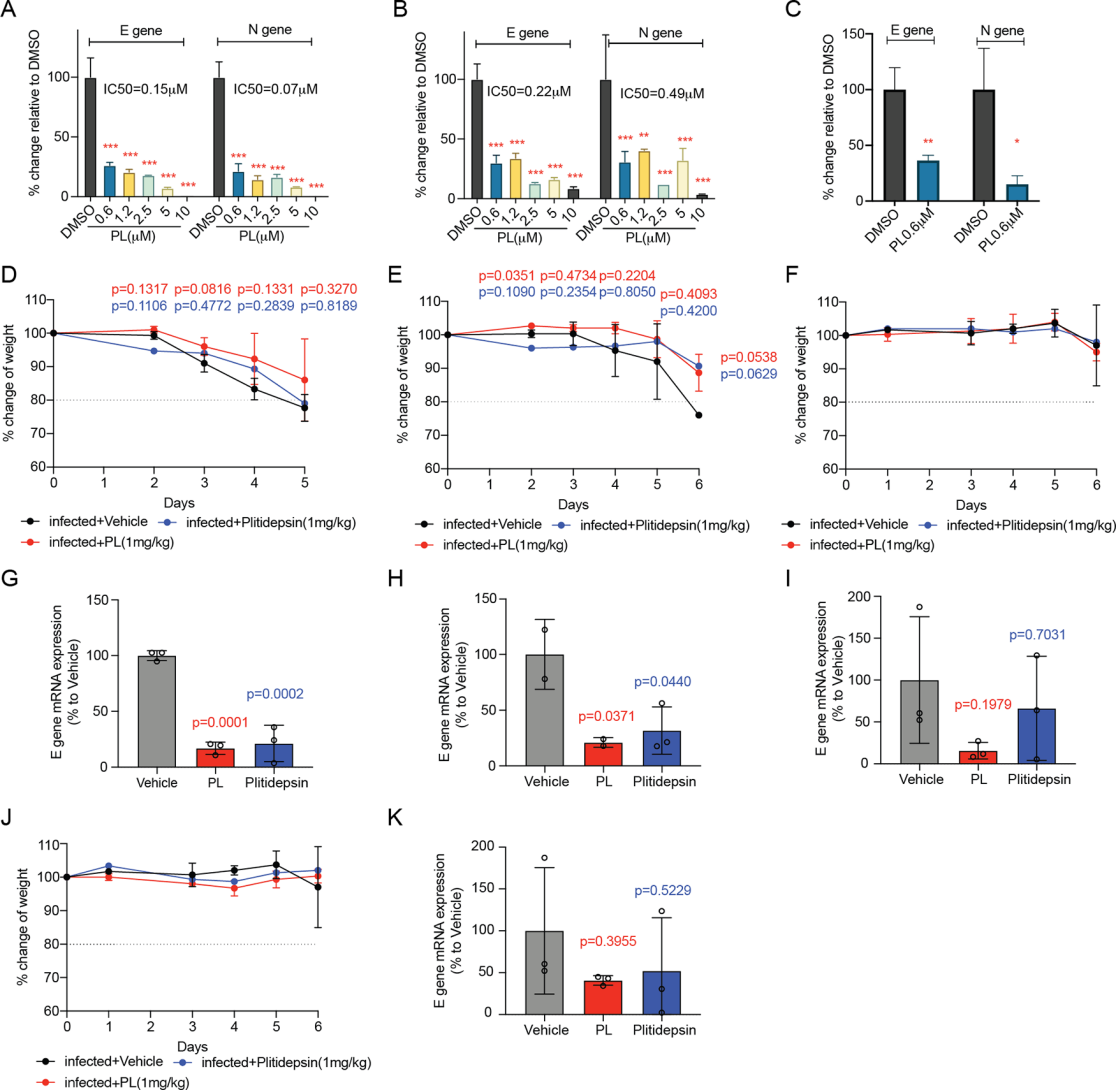
PL inhibits SARS-CoV-2 alpha, delta, and omicron variants infection both *invitro* and *in vivo*. (A–C) *In vitro* antiviral activity of PL in VERO-CCL 81 cells evaluated by the qPCR assay, quantifying E gene and N gene of SARS-CoV-2 virus. Cells were pretreated with PL at indicated concentrations for 1 h, followed by addition and incubation for 1 h of alpha (A), delta (B), or omicron (C) VOCs. Finally, fresh PL media were replaced for another 24 h incubation. IC_50_ values of PL in the VERO-CCL 81 cell line were indicated. Data are presented as mean ± SD. Two-way ANOVA test or unpaired *t* test. *P < 0.05, **P < 0.01, ***P < 0.001. (D–I) In an in vivo prophylactic treatment setting, mice were treated with indicated compound 1 h prior to infection, then challenged with different VOCs. The body weight change curve of vehicle control, PL-, or Plitidepsin-treated mice, infected with alpha VOC (D), delta VOC (E), or omicron VOC (F). The qPCR assay determined lung viral titers on Day 6 for alpha VOC (G), delta VOC (H), and omicron VOC (I), respectively. (J–K) In an in vivo therapeutic treatment setting, mice were treated with indicated compound 1 day after omicron VOC infection. The body weight curve (J) and qPCR assay (K) determined lung viral titers on Day 6. Mean ± SD. Multiple *t* test or one-way ANOVA test.

Given the continuous evolution of the SARS-CoV-2 virus, several new variants emerged. Based on that, we further tested PL against three VOCs in VERO-CCL 81 cells: the alpha (B.1.1.7), the delta (B.1.617.2), and the omicron (B.1.1.529) variants. We observed that PL could inhibit alpha and delta VOCs in a dose-dependent manner with IC_50_ of 0.15 μM for the E gene and 0.07 μM for the N gene for alpha VOC (Figure 3A), 0.22 μM for the E gene and 0.49 μM for the N gene for delta VOC (Figure 3B), respectively, as determined by the qPCR assay. In addition, the qPCR result showed that 0.6 μM of PL can significantly reduce both genes (Figure 3C) of the omicron VOC. Interestingly, we observed that the IC_50_ values of PL are even lower for these variants relative to the ancestral, which suggests PL is more potent against those variants. This observation may result from the lower capacity of VOCs to propagate *in vitro* relative to the ancestral virus.^17^

Moreover, we evaluated the efficacy of PL against SARS-CoV-2 VOCs *in vivo* using the mouse model described previously. The mice were administered with 1 mg/kg PL or Plitidepsin 1 h prior to infection, challenged with 5 × 10^3^ PFU/mouse of alpha VOC or 1 × 10^4^ PFU/mouse of delta VOC or 1.5 × 10^3^ PFU/mouse of omicron VOC on Day 0, and monitored daily until Day 5 or 6 when the vehicle mice reached 80% of initial body weight and showed severe disease signs. At this point, mice were euthanized, and lungs were obtained for virus loading quantification. The body weight curves showed that PL-treated infected mice could slightly delay the body weight loss compared to the vehicle group, both for alpha VOC (Figure 3D) and delta VOC (Figure 3E), while the Plitidepsin-treated group could only delay body weight loss caused by the infection of delta VOC (Figure 3E) but not alpha VOC (Figure 3D). The qPCR assay supported that both PL and Plitidepsin treatment could significantly reduce the lung virus loading after alpha VOC infection (Figure 3G) or delta VOC infection (Figure 3H). Though omicron VOC has limited impact on weight loss and lung infection compared to previous variants shown in K18-hACE2 transgenic mice,^18^ it is critical to evaluate whether PL can act as a pan-variant antiviral. We observed that prophylactic PL treatment does not alter weight loss during omicron infection, neither Plitidepsin (Figure 3F), but PL significantly reduces lung viral load relative to infected vehicle and Plitidepsin groups (Figure 3I). All these data suggest that PL treatment administered prophylactically can effectively protect mice from infection with different SARS-CoV-2 variants.

Considering clinical practice, we recognize that a therapeutic that is effective postinfection is perhaps more urgently required and a more clinically relevant setting. Thus, we established a regimen where mice were only treated with PL or plitidepsin 1 day after infection. In this scenario, neither PL nor Plitidepsin affected weight loss (Figure 3J), but both decreased lung viral loads (Figure 3K). This demonstrates that PL can potentially function as a pan-variant antiviral against developing SARS-CoV-2 VOCs. In addition, prophylactic treatment of PL is an efficacious therapeutic strategy.

### PL Antiviral Activity Is a Result of Selective ROS Induction in Infected Cells

**Figure 4:**
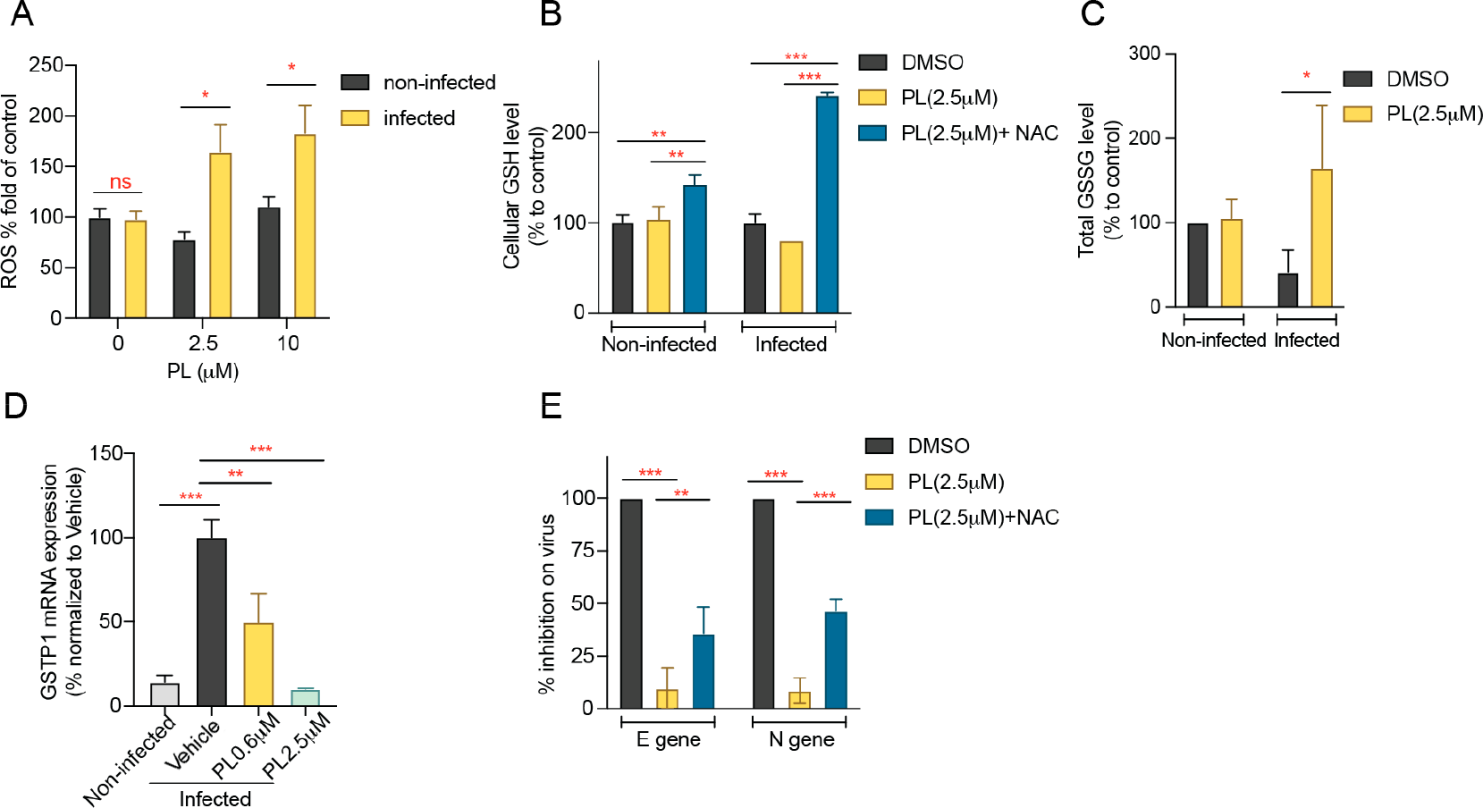
PL selectively induces ROS in infected cells. VERO-CCL 81 cells were pretreated with 5 mM NAC for half an hour, followed by PL treatment for 1 h, then virus inoculum for 1 h. (A) PL induced ROS elevation in infected cells, not noninfected cells. (B–C) PL-mediated modulation of GSH (B) and GSSG (C). (D) GSTP1 mRNA level quantified by the qPCR assay. (E) PL-induced virus inhibition can be rescued by NAC. Mean ± SD. Multiple *t* test or one-way ANOVA test or two-way ANOVA test. *P < 0.05, **P < 0.01, ***P < 0.001.

Given that PL selectively kills cancer cells but not normal cells by inducing ROS,^10^ we investigated if PL could also selectively induce ROS in SARS-CoV-2 infected cells. We first determined the effect of PL on total cellular ROS levels and found that PL can selectively induce ROS in infected cells (Figure 4A). More specifically, we observed that PL treatment slightly decreases reduced glutathione (GSH) levels and increases oxidized glutathione (GSSG) levels in infected cells, yet PL had no effect on GSH or GSSG levels in noninfected cells (Figure 4B–C). GSH is one of the most important ROS scavengers, and the reaction change from GSH to GSSG can serve as an indicator of oxidative stress.^19^ Our results are consistent with previous reports that NAC supplementation increases GSH levels.^20^ Additionally, qPCR results showed that the pi-class glutathione *S*-transferase (GSTP1) was upregulated in infected cells relative to noninfected ones. We found that PL treatment reduced GSTP1 levels (Figure 4D). GSTP1 is known as a protein that regulates oxidative stress. Our data suggest that overexpressed GSTP1 in infected cells can be targeted by PL, which provides an explanation for the selectivity of PL in infected cells. The decreased expression of GSTP1 inhibited by PL leads to ROS accumulation and GSH reduction in infected cells. To further prove the specificity of PL and the importance of the two reactive olefins of PL for ROS elevation, we tested a fully saturated derivative of PL, namely, PL22. We observed that PL22 lost the antiviral activity due to lack of C2-C3 and C7-C8 olefins, which supports the fact that the two olefins of PL display an important role for its antiviral activity (Figure S2). Moreover, cotreatment with PL and the ROS inhibitor *N*-acetyl-l-cysteine (NAC, 5 mM) attenuated PL-mediated GSH depletion (Figure 4B). The increased reliance of infected cells on the ROS stress-response system may underlie the antiviral action generated by PL. To prove this hypothesis, cells were treated with NAC prior to PL treatment and virus inoculation. We showed that PL-induced virus inhibition is rescued by the antioxidant NAC (Figure 4E), suggested by both the E gene and N gene of SARS-CoV-2 quantification by qPCR. Altogether these observations indicate that the antiviral activity is mediated by PL inhibition of GSTP1, which leads to selective accumulation of ROS in infected cells. This MOA supports that PL acts as a host-directed antiviral against SARS-CoV-2 virus.

### PL Potently Triggers the MAVS-Induced IFN-JAK-STAT Pathway

**Figure 5:**
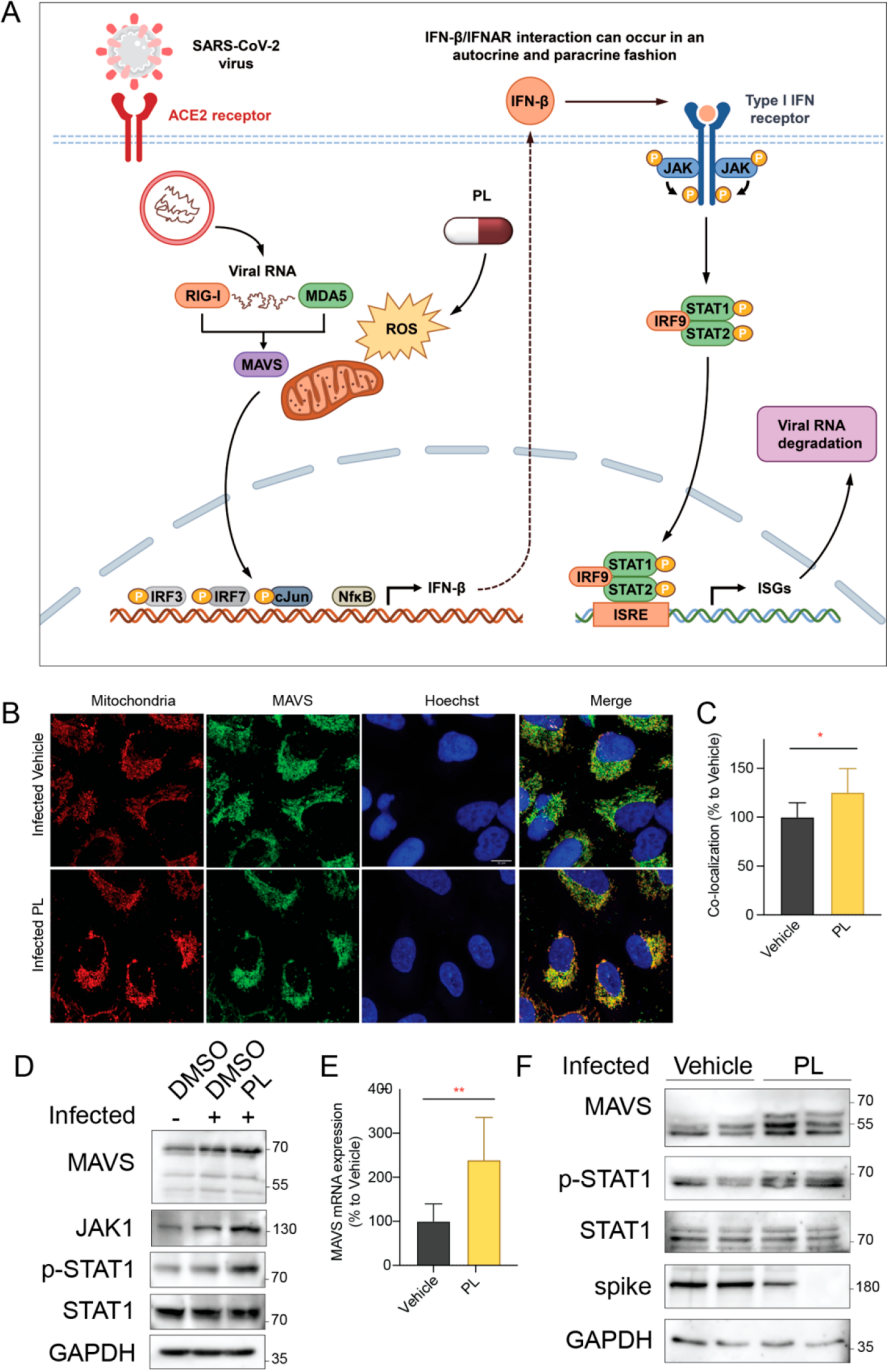
PL triggers the JAK/STAT pathway through the activation of MAVS. (A) Antagonism of interferon signaling by SARS-CoV-2 and potential antiviral mechanism of PL. (B) Fluorescence microscopy analysis of MAVS colocalization with mitochondria in A549-hACE2 cells, treated with 0.6 μm PL for 1 h prior to infection. MAVS colocalization with mitochondria is indicated by the yellow staining in the merged image and was quantified using ImageJ software (C). Blue - Hoechst-33342; green - MAVS; red - citrate synthase, 63× amplification, scale bar 10 μm. (D) Western blot analysis of MAVS, JAK1, and p-STAT1 proteins in A549-hACE2 cells, treated with PL for 1 h prior to infection. GAPDH expression was used as a loading control. (E) qPCR analysis of the MAVS mRNA level in A549-hACE2 cells, treated with PL for 1 h prior to infection. (F) Western blot analysis of MAVS, p-STAT1, and spike proteins from lung extracts of transgenic K18-hACE2 mice treated with 1 mg/kg PL or vehicle control 1 h prior to infection. Mean ± SD. Unpaired *t* test, *P* < 0.05, **P < 0.01.

ROS modulates various inflammatory processes, and it is reported that increased cellular ROS amplifies retinoic acid-inducible gene 1 (RIG-1) signaling and mitochondrial antiviral-signaling protein (MAVS) function.^21^ Upon reaching the cytoplasm, coronavirus RNA is detected by the cytoplasmic RNA sensors RIG-1 and melanoma differentiation-associated protein 5 (MDA5), which triggers conformational changes in these sensors and results in an interaction with MAVS, which in turn recruits the downstream effector proteins, including the IFN-JAK-STAT pathway, for further antiviral response (Figure 5A).^22^ Fluorescence microscopy showed that the percentage of MAVS colocalization with mitochondria increased in PL-treated infected cells, indicating MAVS activation by PL (Figure 5B–C). Then a Western blot assay was performed to quantify the protein levels *in vitro* and to test whether the antiviral effect of PL is related to the above pathway. We observed that MAVS, JAK1, and p-STAT1 proteins are upregulated in PL-treated infected cells compared to control DMSO-treated infected cells (Figure 5D). The upregulation of the MAVS RNA level could also be validated by the qPCR assay (Figure 5E). A similar changing pattern in protein levels was detected *in vivo*. We tested the protein levels using the homogenized lung samples from vehicle- and PL-treated infected mice and found that MAVS and p-STAT1 are upregulated in PL-treated mice compared to nontreated mice. In contrast, the SARS-CoV-2 spike protein level decreases, which further demonstrates PL-mediated reduction of virus infection (Figure 5F). Taken together, these results support that the antiviral activity of PL is related to the MAVS-induced IFN-JAK-STAT pathway, eventually leading to the viral degradation.

## Discussion

In this study, we describe the *in vitro* and *in vivo* antiviral activity of PL, a small molecule natural product isolated from the long pepper (*Piper longum* Linn). The potential for widespread antiviral action against SARS-CoV-2 makes PL a candidate for further preclinical and eventually clinical studies for COVID-19. Importantly, PL acts as a host-targeted antiviral by selectively inducing ROS in infected cells and not in noninfected cells, making it more resistant to naturally occurring viral variants, which contrasts with viral-targeted treatments.

The K18-hACE2 mouse model used in this study is reliable for simulating COVID-19 pathophysiology and evaluating antiviral countermeasures.^23^ In this model, SARS-CoV-2 infection causes severe viral replication in the lung, leading to severe immune cell infiltration, inflammation, and lung damage, which is extremely useful for evaluating the antiviral or anti-inflammatory efficacy of compounds.^23^ Using this severe model of COVID-19, PL significantly delayed the onset of clinical signs and disease progression, which was accompanied by a marked reduction in lung viral load. We directly compared the activity of PL with the benchmark HDA molecule, Plitidepsin, and showed three critical advantages of PL over plitidepsin: (1) while the effect of Plitidepsin on mortality was previously not reported,^11^ our data show that PL has a superior effect relative to Plitidepsin with regard to mice protection from overt disease and weight loss; (2) PL treatments lead to significant reduction of viral titers in the lung relative to Plitidepsin; and finally (3) while both molecules act as host-directed antivirals, PL can be safely administered intranasally, while Plitidepsin administration relies on subcutaneous and intravenous injections in mice and patients, respectively. The intranasal route is believed to display a superior advantage because it can induce both mucosal and systemic immune responses,^24^ which eliminates the shortcomings of current vaccines. Importantly, through PL formulation, a self-administration drug may be achieved, which would decrease the requirement for professional healthcare labor and increasing patient compliance. Our studies provide strong evidence for the safety and superior potency of PL as an HDA drug relative to Plitidepsin. PL acts as a pan-SARS-CoV-2 drug being efficacious against alpha, delta or omicron VOCs.

As a biologically active alkaloid/imide from long pepper, which is widely used in Ayurvedic medicine, PL was shown to exert various pharmacological activities.^8^ A recent study reported that PL reduces systemic and pulmonary inflammatory alterations, presenting a prospective therapeutic for reducing the inflammation generated by cigarette smoke,^25^ which supports our observations that PL has the capacity to decrease lung inflammation.

Concerning the MOA of PL antiviral activity, we showed that PL selectively induces ROS in infected cells but not in noninfected cells. This antiviral selectively pattern is dependent on PL targeting and inhibiting GSTP1, which is upregulated in infected cells and leads to induction of ROS levels. GSTP1 was reported to display a critical role in COVID development, as evidenced by the fact that carriers with variant GSTP1- *Val allele* exhibit lower odds of COVID-19 infection.^26^ Another analysis also proposed that Clomipramine can inhibit coronavirus by interacting with GSTP1.^27^ Notably, a structure–activity study revealed that PL binds to GSTP1 via its two olefins, which inhibits GSTP1, induces ROS, and leads to cancer cell apoptosis.^28^ Our observations that PL inhibits GSTP1 in infected cells leading to ROS accumulation, and that both olefins are required for this activity, are consistent with those reports. ROS are potent regulators of MAVS, having a direct effect by promoting MAVS signaling complex. In fact, oxidative stress promotes MAVS oligomerization and consequently type I IFN secretion, independently of the cytosolic sensor RIG-I.^29^ Since PL induces ROS selectively in infected cells through GSTP1 inhibition, it can modify innate immune signaling through direct MAVS activation. The cell innate immune system is regulated by MAVS and its downstream effectors. Several viral-encoded peptides have been reported to localize to mitochondria and interfere with MAVS. Upon sustained infection, MAVS can be targeted for proteolysis leading to its degradation, loss of MAVS clusters from mitochondrial surface, and release to the cytosol.^30,31^ Concerning SARS-CoV-2 infection, several reports have shown a direct interaction with MAVS with consequent impairment of the innate immune response pathways. SARS-CoV-2 M,^32^ ORF9b,^33^ and ORF10^34^ peptides were found to associate with MAVS, inhibiting its accumulation and aggregation to facilitate viral replication.

The ROS accumulation explains how PL upregulates MAVS expression and activates the IFN-JAK-STAT pathway. This pathway is essential in regulating local and systemic inflammation in response to viral infections. JAKs are responsible for the phosphorylation and activation of STATs. STAT1, after phosphorylation, forms a complex with other proteins and translocates to the nucleus, where it interacts with the interferon-stimulated response element (ISRE) promoter to increase the production of interferon-stimulated genes (ISGs) that conduct various antiviral roles.^22^ SARS-CoV-2 is believed to desensitize host cells to interferon by inhibiting the JAK-STAT pathway,^35^ and therefore PL may provide a therapeutic opportunity to resensitize the host cells’ response to interferon by activating ROS-related MAVS protein. In addition, the selectivity of PL in inducing ROS in infected cells, but not in normal cells, provides the safety of PL treatment and on-target specificity.

Collectively, we identified and validated PL as an effective host-directed antiviral compound against SARS-CoV-2 and VOCs by selectively inducing ROS in host infected cells and further triggering the MAVS-induced IFN-JAK-STAT pathway. In the future, we expect to evaluate PL against SARS-CoV-2 infection via pharmacokinetics and immunogenicity characterization and move this study to practical use in addition to current antiviral strategies against emerging variants.

## Methods

### Cell Lines and Culture

VERO-CCL 81 cells and human lung adenocarcinoma epithelial A549 cells expressing hACE2 cells were cultured in Dulbecco’s modified Eagle’s medium (DMEM, Life Technologies), supplemented with 10% (v/v) fetal bovine serum (FBS), 1% penicillin-streptomycin, and 1% glutamax (ThermoFisher). All cell lines were cultured at 37 °C and 5% CO_2_.

### Viral Strains and Stocks

The Wuhan-like early European SARS-CoV-2 B.1 lineage was isolated from a Portuguese patient (internal reference: 606_IMM ID_5452) at approximately 1.7 × 10^6^ PFU/mL. The alpha variant (NR-54000; lineage B.1.1.7, Isolate hCoV-19/England/204820464/2020) was obtained through BEI Resources, NIAID, NIH, contributed by Bassam Hallis. The delta variant (NR-55611; lineage B.1.617.2; Isolate hCoV-19/USA/PHC658/2021) was obtained through BEI Resources, NIAID, NIH, contributed by Dr. Richard Webby and Dr. Anami Patel. The omicron variant (lineage B.1.1.529) was obtained through the WHO BioHub System. The original and variants virus stocks were propagated using VERO-CCL 81 cells, 1.4 × 10^7^ cells were seeded in several T175 culture flasks and infected the following day at a 0.005 multiplicity of infection (MOI) in 10 mL of maintenance medium (DMEM medium supplemented with 2.5% FBS, 1% penicillin-streptomycin and 1% glutamax). After 1 h of inoculation, the culture medium was replaced with another fresh 25 mL of maintenance medium, and virus propagation was continued until 4-day postinfection. Cell supernatants were collected from the T175 flasks to isolate the virus, centrifuged at 300*g* for 5 min to remove the cell debris, aliquoted, and stored at −80 °C. The titers of the generated stock virus were quantified by plaque assay. All work with infectious SARS-CoV-2 was conducted at a Level 3 Biosafety Laboratory (BSL3) facility of Instituto de Medicina Molecular, where all procedures follow Directive 2000/54/EC - on the protection of workers from risks related to exposure to biological agents at work, Directive (EU) 2020/739 - as regards the inclusion of SARS-CoV-2 in the list of biological agents known to infect humans, and World Health Organization (WHO) guidelines.

### SARS-CoV-2 Infection Experiments

All antiviral experiments were conducted in a Biosafety Level 3 (BSL3) animal facility at the Institute of Molecular Medicine (iMM) in Lisbon, Portugal. VERO-CCL 81 cells or A549-hACE2 cells were seeded in 24 well plates at 1.6 × 10^5^/well the day before infection. A series of concentrations of PL was added 1 h prior to or after infection. The SARS-CoV-2 virus was thawed, vortexed, centrifuged, and used to infect the cells at different multiplicities of infection (MOI), 0.035 of MOI for VERO-CCL 81 cells and 0.1 of MOI for A549-hACE2 cells. After 1 h inoculation, the inoculum was replaced by fresh PL media. Supernatants were collected for the plaque assay after 24 h treatment, and cells were collected for qPCR quantification to quantify virus load.

### Plaque Assay (Detect Viral Plaque-Forming Units)

VERO-CCL 81 cells were seeded in 6 well-plates at 8 × 10^5^/well and allowed to grow to around 80% confluence after 24 h. Supernatants of cultures treated with compounds were 1:10 serially diluted in maintenance medium to obtain 10^–1^ and 10^–2^ dilutions, added to preseeded 6-well plate cells, and incubated at 37 °C for 1 h, shaken every 15 min. Then they were replaced with 1.25% carboxymethylcellulose (CMC) and incubated at 37 °C for 4 days. The CMC was removed after the incubation, and cells were fixed with 4% formaldehyde/PBS and stained with 0.1% toluidine blue. Viral plaques were counted to determine the infectious titers [PFU (plaque forming units)/mL].

### Cytotoxicity Assay

VERO-CCL 81 cells or A549-hACE2 cells were seeded in 96 well-plates at 1 × 10^4^/well the day before treatment. An increasing concentration of PL was added to cells for another 24 h incubation. CellTiter Blue viability assay (Promega, Cat#G8080) was used to assess the viability of cells after treatment, according to the manufacturer’s protocol.

### Viral RNA Isolation and Quantitative PCR

The viral pellet was suspended in NVL buffer and extracted using the NZY Viral RNA Isolation Kit (NZYtech, Cat#MB40701). 1 μg of total RNA was used for RT-PCR using the NZY First-Strand cDNA Synthesis Kit (NZYtech, Cat#MB12502). The qPCR amplification was performed with a dilution of 1:10 of cDNA using the iTaq Universal SYBR Green Supermix (Biorad, Cat#1726124) according to the manufacturer’s instructions and analyzed on QuantStudio 5 real-time PCR machine (Applied Biosystems). The relative quantification of target gene expression was performed using the comparative cycle threshold (C_T_) method. The primer sequences are listed in Table 1 below.

**Table 1:** Primer Sequences Used for Quantitative PCR

gene	forward	reverse	ref
SARS-CoV-2 E gene	ACAGGTACGTTAATAGTTAATAGCGT	ATATTGCAGCAGTACGCACACA	(36)
SARS-CoV-2 N gene	GACCCCAAAATCAGCGAAAT	TCTGGTTACTGCCAGTTGAATCTG	(37)
18S	GTAACCCGTTGAACCCCATT	CCATCCAATCGGTAGTAGCG	
MAVS (human)	GTGCCTACTAGCATGGTGCTC	GACCCAAGGCCCCTATTCT	
GSTP1 (human)	GGAGACCTCACCCTGTACCA	GTCCTTCCCATAGAGCCCAAG	

### Western Blotting Analysis

For cell lysis analysis, cells were lysed using whole cell lysis buffer (50 mM Tris-HCl pH = 8.0, 450 mM NaCl, 0.1% NP-40, 1 mM EDTA), supplemented with 1 mM DTT, protease inhibitors (Sigma), and phosphatase inhibitors (Sigma). For in vivo experiments, the left lung of mice was homogenized in 3 mL of DMEM, and 750 μL was transferred to an equal volume of whole cell lysis buffer, supplemented as above. Protein concentrations were quantified using Bradford Assay (Biorad). Thirty μg of proteins were loaded per lane and separated on SDS-PAGE gels, and then transferred onto polyvinylidene difluoride (PVDF) membranes (GE Healthcare). Membranes were blocked for 1 h with 5% skim milk or 5% BSA in TBS supplemented with 0.05% Tween-20 (TBST) at room temperature for 1 h and then probed with any of the following specific primary antibodies in 5% skim milk or BSA at 4 °C overnight. After three times washing with 0.05% TBST, secondary antibodies, antimouse (1:5000) or antirabbit (1:5000) (Jackson ImmunoResearch), were added to the membrane in 0.05% TBST for 1 h at room temperature. All membranes were washed three times and exposed using ECL substrate (Biorad, Cat#170-5060) and Amersham 800 Imaging System (Cytiva). The primary antibodies included MAVS (sc-166583), JAK1 (sc-376996), phos-STAT1 (sc-8394), STAT1 (sc-464), GAPDH (sc-47724), goat antimouse IgG H&L (HRP) (Abcam, ab205719), and goat antirabbit HRP (Abcam, ab6721).

### Animal Model of SARS-CoV-2 *in Vivo* Experiments

Animal studies were conducted in the BSL-3 Facility strictly with the relevant EU and national legislation, approved by the Portuguese official veterinary department for welfare licensing – Direção Geral de Alimentação e Veterinária – (license number 01878/2021) and the Instituto de Medicina Molecular Animal Ethics Committee. Eight to 12-week-old specific pathogen-free hemizygous for Tg(K18-ACE2)2Prlmn mice (Strain B6.Cg-Tg(K18-ACE2)2Prlmn/J, the Jackson laboratory strain 034860) were used in this study. Mice were treated with Vehicle (*n* = 8), PL at 1 mg/kg (*n* = 18) or Plitidepsin at 1 mg/kg (*n* = 6) 1 h prior to infection. For alpha and delta variants in vivo study, 9 mice were used for each variant study, including 3 for vehicle, 3 for PL and 3 for Plitidepsin treated 1 h before infection. For omicron variant, 18 mice were used in total including half for prophylactic study (treated 1 h prior to infection) and another half for therapeutic treatment (treated 1 day after infection), with vehicle (*n* = 3), 1 mg/kg PL (*n* = 3) or 1 mg/kg Plitidepsin (*n* = 3) for each setting. Vehicle and PL were delivered by intranasal instillation, and Plitidepsin was injected subcutaneously according to previous publication.^11^ Intranasal inoculation with 1 × 10^4^ PFU of SARS-CoV-2 or delta VOC, or 5 × 10^3^ PFU of alpha VOC, 1.5 × 10^3^ PFU of omicron VOC, in 50 μL of Maintenance medium, was then performed. Mice were monitored daily for body weight of mice and clinical signs of disease. On Day 5 postinfection, all mice were humanely euthanized, and lung was harvested. Left lung was homogenized by ULTRA-TURRAX Tube Drive control and frozen at −80 °C for viral quantification by qPCR and plaque assay and protein analysis. Right lung was fixed in 10% neutral buffered formalin and submitted for histopathology. Previously to the infection experiments, one group of 3 noninfected mice were treated with 1 mg/kg PL IN to test for weight loss or other clinical signs of disease.

### Mouse Lung Histological Analysis

Lungs were fixed in formalin, embedded in paraffin, and tissue sections cut at and stained with H&E. Scoring of lung pathology was performed taking into account features described in Table 2, adapted from previously published criteria.^38^ Briefly, hemorrhage, congestion, necrosis, and hyperplasia of bronchiolar epithelium, inflammation, and proteinaceous debris were scored according to a 5-tier scale: 0, absent; 1, minimal; 2, mild; 3, moderate; 4, marked. For scoring thickness of alveolar wall: 0, < 2×; 1, 2–4×; 2, 5–10×; 3, 11–20×; 4, no airspace. For percentage of area affected: 0, none; 1, < 25% of total lung area; 2, 26–50%; 3, 51–75%; 4, > 76%. The final score for each lung/animal was calculated by dividing the final score per number of features assessed (total of 8) and multiplying by % of the area affected (multiplication factor for score 1 was 0,25; for 2, 0,5; for 3, 0,75 and for 4, corresponding to 76 to 100% of lung area affected, multiplication factor was 1). Images were acquired in the Hamamatsu NanoZoomerSQ, using NDP.view2 software (Hamamatsu).

**Table 2:** Scoring of Lung Pathology^a^

		score per feature				
features		0	1	2	3	4
A	hemorrhage	absent	minimal	mild	moderate	marked
B	congestion					
C	necrosis bronchiolar epithelium					
D	hyperplasia bronchiolar epithelium					
E	inflammation, interstitial					
F	inflammation, alveoli					
G	proteinaceous debris in airspace					
H	thickening of the alveolar wall	<2×	2–4×	4–10×	10–20×	no airspace
I	area affected (%)	none	<25	26–50	51–75	>76

a Final score = [(A + B + C + D + E + F + G + H)/(number of features, 8)] × I.

### Measurement of ROS Level

The level of reactive oxygen species (ROS) was measured using the ROS-ID Total ROS detection kit (Enzo, Cat#ENZ-51011), according to the manufacturer’s instructions. Briefly, VERO-CCL 81 cells were seeded in 96-well black wall/clear bottom plates at a density of 1 × 10^4^ cells per well 1 day before the assay. Cells were treated with PL for 1 h prior to the infection, and then inoculum along with the ROS detection reagent at 37 °C for another 1 h. Subsequently, the ROS level was measured using a fluorescence microplate reader (TECAN Infinite M200, Switzerland) and standard fluorescein (*E*_x_ = 488 nm, *E*_m_ = 520 nm) filter set. The assay used *N*-acetyl cysteine (NAC, 5 mM) as a ROS inhibitor.

### Glutathione (GSH) and Glutathione Disulfide (GSSG) Levels

Measurement of GSH/GSSG was performed following Rahman et al., with minor adjustments. Cells were seeded in a 6-well plate at a density of 4.5 × 10^5^ cells per mL and subjected to different treatments and inoculations. During harvesting, cells were collected in ice-cold 1× PBS, centrifuged, and resuspended in ice-cold extraction buffer (0.1% Triton-X and 0.6% sulfosalicyclic acid in KPE). After homogenization, samples were sonicated in ice for 3 min and subjected to 2 freeze–thaw cycles to ensure proper lysis. Then the samples were centrifuged at 3000*g* for 4 min at 4 °C, and the supernatant was collected and stored at −80 °C until further procedures. For GSH determination, 20 μL of each sample was mixed with freshly prepared DTNB and glutathione reductase, incubated for 30 s, and mixed with β-NADPH. Absorbance was measured at 412 nm for 2 min to determine the rate of 2-nitro-5-thiobenzoic acid formation. For GSSG quantification, 100 μL of the sample was incubated with 2 μL of 2-vinylpyridine for 1 h at room temperature. For neutralization, 6 μL of triethanolamine was added and incubated for 10 min. GSSG samples were then subjected to the same protocol as for the GSH determination. The GSH and GSSG levels were determined from comparisons with a linear GSH or GSSG standard curve, respectively.^39^

### Fluorescence Microscopy

The day before the beginning of the experiment, A549-hACE2 cells were seeded at a confluence of 3 × 10^4^ per well in a μ-Slide 8-well Ibidi plate. Subsequently, cells were treated with PL 1 h before infection, followed by SARS-CoV-2 inoculation at a 0.1 MOI for another hour. Then the medium was replaced with fresh maintenance medium containing PL for 24 h. The next day, cells were washed with 1× PBS and fixed with 4% paraformaldehyde in 1× PBS for 30 min, and then washed three times with 1× PBS. After fixation, cells were permeabilized with 0.3% triton in 1× PBS for 10 min, washed three times with 1× PBS, and blocked in 3% BSA in PBS for 1 h at RT. Cells were then incubated with the first antibody MAVS (Santa Cruz, sc-166583) and citrate synthase (Proteintech, #16131-1-AP) in blocking buffer for 3 h at RT, followed by secondary antibody (AF-488 and AF-568, respectively) in 1× PBS for 1 h at RT. Nuclei were stained with Hoechst 33342 for 10 min. Images were acquired in z-stacks with the 63× objective of the LSM880 Airyscan setup (Zeiss) and processed and analyzed in ImageJ, using JaCoP Macro.^40^

### Data Analysis

The data quantification was performed using the GraphPad Prism software. Data are presented as mean ± SD. One-way or two-way ANOVA was used to compare differences in more than two groups. A Student’s *t* test was used to compare differences between two groups. In all circumstances, *P*-values ≤ 0.05 were considered significant (*P < 0.05, **P < 0.01, ***P < 0.001).

## Supporting Information Available

Representative histopathology microphotographs for other organs (Figure S1); the fully saturated derivative of PL (Figure S2); chemical synthesis and characterization (PDF)
