# Supplementary material for: A Selective SARS-CoV-2 Host-Directed Antiviral Targeting Stress Response to Reactive Oxygen Species
Source: ACS Cent Sci. 2023 Jan 13;9(1):109–21. doi: 10.1021/acscentsci.2c01243 (PMC9881195; doi:10.1021/acscentsci.2c01243)
Supplement: Supplementary file 1 — oc2c01243_si_001.pdf [file oc2c01243_si_001.pdf]

Supporting Information

A selective SARS-CoV-2 host-directed antiviral targeting stress response to ROS

Cong Tang,*#1 Ana R Coelho,#1 Maria Rebelo,#1 Hannah Kiely-Collins,[2] Tânia Carvalho,[3] Gonçalo J. L. Bernardes*[1,2]

*Correspondence should be addressed to G.J.L.B. and C.T.:
E-Mail: gbernardes@medicina.ulisboa.pt and cong.tang@medicina.ulisboa.pt

[1] Instituto de Medicina Molecular João Lobo Antunes, Faculdade de Medicina, Universidade de Lisboa, Avenida Professor Egas Moniz, 1649-028, Lisboa, Portugal

[2] Yusuf Hamied Department of Chemistry, University of Cambridge, Lensfield Road, Cambridge CB2 1EW, UK

[3] Champalimaud Foundation, Avenida de Brasília, 1400-038, Lisboa, Portugal

#These authors contributed equally.

Table of Contents

Figure S1 Representative histopathology microphotographs — Page S2
Figure S2 The fully saturated derivative of PL — Page S3
Chemical Synthesis and Characterization — Page S4
References — Page S17

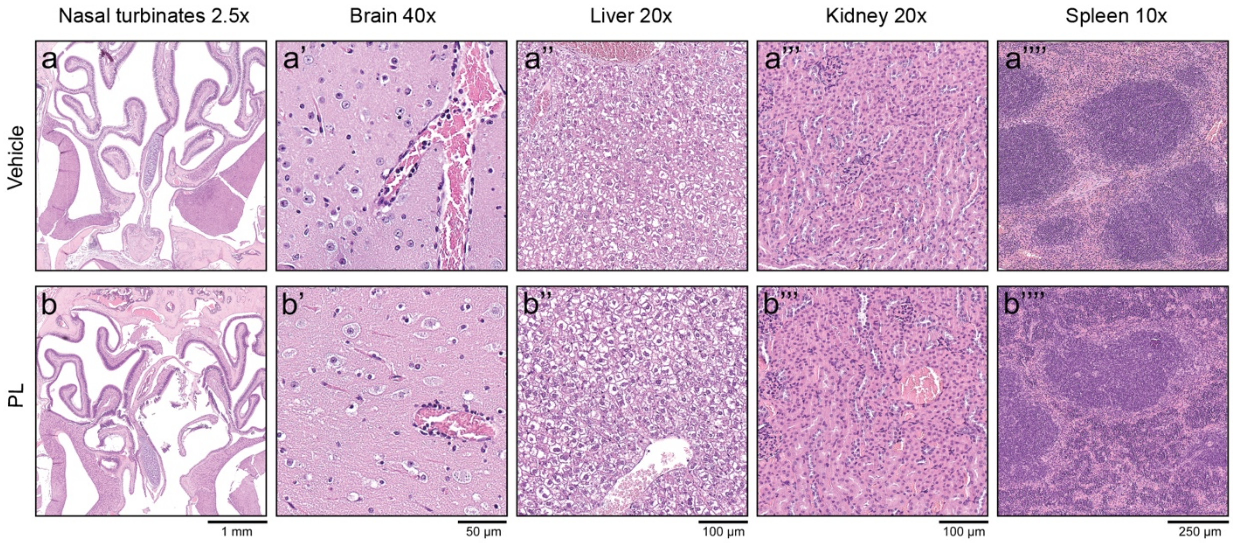

Figure S1. Representative microphotographs of nasal mucosa, brain, liver, kidney and spleen, from mice infected with SARS-CoV-2 at 5 days post-infection, untreated (vehicle) and treated with PL.

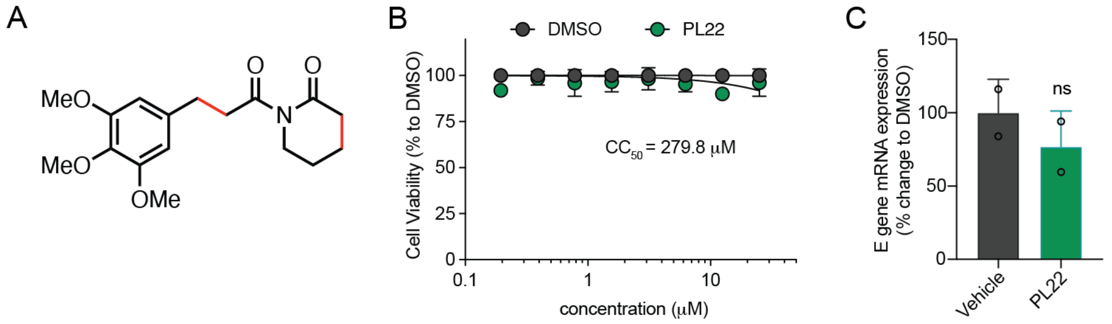

Figure S2. The fully saturated derivative of PL. (A) Chemical structure of PL derivative PL22. (**B**) Cytotoxicity of PL22 on VERO-CCL 81 cell line. (**C**) *In vitro* antiviral activity test of 0.6 μm PL22 by quantifying E gene of SARS-CoV-2 by qPCR assay. Mean±SD. Unpaired T-test.

Chemical Synthesis and Characterization

Synthetic chemistry methods

All non-aqueous reactions were performed in oven-dried glassware under a dry argon or nitrogen atmosphere with dry solvents unless otherwise stated. Reaction vessels were heated using thermostatically controlled DrySyn blocks with the liquid level of the flask below that of the heating block. Reaction temperatures refer to the thermostat set-point. A reaction temperature of 0 ºC refers to an external ice/water slurry cooling bath. A reaction temperature of -20 ºC refers to an external dry ice/ethylene glycol slurry cooling bath. A reaction temperature of -78 ºC refers to an external dry ice/acetone slurry cooling bath. All reagents were purchased from commercial sources and used without further purification unless otherwise stated. CH_2Cl_2, THF and Et_2O were purified either according to the method of Grubbs and Pangborn[1] or by distillation under an inert atmosphere (CH_2Cl_2, MeOH and MeCN were distilled from calcium hydride. THF and Et_2O were pre-dried over sodium wire then distilled from calcium hydride and lithium aluminium hydride). Petroleum ether, *n*-hexane and EtOAc were distilled on site. 'Petrol' refers to the distillate of petroleum ether collected between 40–60 ºC unless otherwise stated. Water used experimentally was deionised and prepared on site.

Flash column chromatography was performed using Merck silica gel 60 Å (40 – 63 µm). Analytical thin layer chromatography was performed using Merck Silica gel 60 F254 1 mm glass plates and visualised by UV (254 nm) or by staining with an indicated solution prepared by known procedures.

NMR spectra were recorded on 400 MHz Avance III HD, 400 MHz Neo 400 and 600 MHz Avance 600 BBI spectrometers. Chemical shifts are reported in parts per million (ppm)

and the spectra are calibrated to the residual solvent peak (^{1}H NMR: $CDCl_3$ δ 7.26 ppm; ^{13}C NMR: $CDCl_3$ δ 77.16 ppm). Multiplicities are described as s (singlet), d (doublet), t (triplet), q (quartet), m (multiplet), dd (double doublet) etc. Coupling constants (*J*) are reported in hertz (Hz) to 1 decimal place using Mestrenova software version 12.0.0 for signal processing. The centre of each peak is reported except for multiplet signals where a range of ppm values are given. High-resolution mass spectra (HRMS) were obtained with an Agilent 1260 Infinity II system.

***N*-(but-3-en-1-yl)acrylamide (1)**

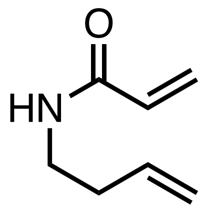

Triethylamine (2.94 mL, 21.1 mmol, 1.5 equiv.) was added to a stirred solution of but-3-en-1-amine (1.00 g, 14.1 mmol, 1.0 equiv.) in CH_2Cl_2 (20 mL) at 0 ºC. Acryloyl chloride (1.37 mL, 16.9 mmol, 1.2 equiv.) was added and the mixture was stirred at room temperature for 3 h. The reaction mixture was diluted with water, and then extracted into CH_2Cl_2 (2 × 10 mL). The solvent was removed *in vacuo* and the crude product was purified by flash column chromatography on silica gel (98:2 CH_2Cl_2/MeOH) to yield **1** as a yellow oil (1.10 g, 14.1 mmol, 63%).[2]

R_f 0.3 (98:2 CH_2Cl_2/MeOH)

^{1}H NMR (500 MHz, $CDCl_3$) δ 6.27 (ddd, *J* = 17.0, 1.5, 0.6 Hz, 1H), 6.07 (ddd, *J* = 17.0, 10.3, 0.6 Hz, 1H), 5.78 (ddt, *J* = 17.1, 10.2, 6.8 Hz, 1H), 5.63 (dt, *J* = 10.3, 1.1 Hz, 1H), 5.16 – 5.06 (m, 2H), 3.42 (tdd, *J* = 6.7, 5.7, 0.9 Hz, 2H), 2.30 (qt, *J* = 6.7, 1.3 Hz, 2H).

^{13}C NMR (126 MHz, $CDCl_3$) δ 165.6, 135.3, 131.0, 126.5, 117.5, 38.6, 33.8.

HRMS *m/z* (ESI+) $C_7H_{11}NO$ ([M+H]+) calculated 125.0841, found 125.0845

5,6-dihydropyridin-2(1*H*)-one (2)

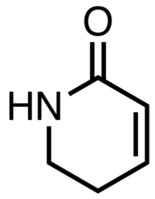

Grubbs-II catalyst (21 mg, 5 mol %) was added to a solution of *N*-(but-3-en-1-yl)acrylamide (0.0625 g, 0.5 mmol) in CH_2Cl_2 (80 mL) and refluxed for 6 h under inert conditions. The mixture was stirred for an additional 1 h at room temperature in open air to deactivate the catalyst. The reaction mixture was filtered through celite, concentrated, and the residue was purified by flash column chromatography on silica gel (1:4 hexane/EtOAc flushed with MeOH/CH_2Cl_2) to give **2** as a brown oil (26.62 mg, 0.27 mmol, 55%).

R_f 0.10 (1:4 Petrol/EtOAc)

^{1}H NMR (400 MHz, $CDCl_3$) δ 6.63 (dt, *J* = 10.0, 4.2 Hz, 1H), 6.40 – 6.27 (m, 1H), 5.88 (d, *J* = 9.9 Hz, 1H), 3.48 – 3.37 (m, 2H), 2.33 (tdd, *J* = 6.9, 4.2, 1.8 Hz, 2H).

^{13}C NMR (101 MHz, $CDCl_3$) δ 166.6, 141.7, 124.8, 39.7, 23.9.

(*E*)-1-(3-(3,4,5-trimethoxyphenyl)acryloyl)-5,6-dihydropyridin-2(1*H*)-one (PL)

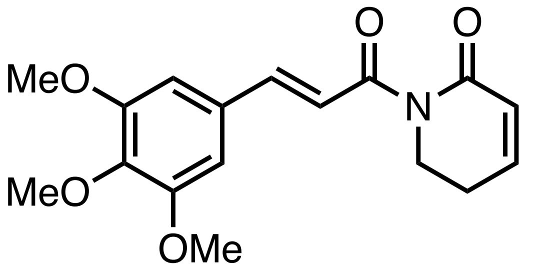

To a solution of (*E*)-3-(3,4,5-trimethoxyphenyl)acrylic acid (104.25 mg, 0.44 mmol, 1 equiv.), in freshly distilled THF (2.5 mL) was added triethylamine (0.05 mL, 0.36 mmol, 0.82 equiv.). Pivaloyl chloride (41.6 mg, 0.35 mmol, 0.79 equiv.) was added at −20 °C and the reaction mixture was stirred for 45 min. To a separate solution of **2** (50.9 mg, 0.525 mmol, 1 equiv.) in freshly distilled THF (2.5 mL) was added *n*-BuLi (0.4 mL, 0.63 mmol, 1.2 equiv.) at −78 ºC under argon and the reaction was stirred for 45 min. Then, anhydride prepared from the above step was added and the reaction mixture was stirred for 1 h. The reaction mixture was quenched with saturated NH_4Cl (1 mL), extracted with ethyl acetate (2 × 5 mL), the organic layer was separated and washed with sat. NaCl (2 × 3 mL) and dried over anhydrous Na_2SO_4. The residue was evaporated *in vacuo* to give a crude product which was purified by column chromatography on silica gel (3:2 Petrol/EtOAc) to yield **PL** as an off-white powder (86.6 mg, 0.27 mmol, 62%).

R_f 0.19 (3:2 Petrol/EtOAc).

^{1}H NMR (400 MHz, $CDCl_3$) δ 7.67 (d, *J* = 15.5 Hz, 1H), 7.42 (d, *J* = 15.5 Hz, 1H), 6.94 (dt, *J* = 9.7, 4.2 Hz, 1H), 6.80 (s, 2H), 6.04 (dt, *J* = 9.7, 1.8 Hz, 1H), 4.03 (t, *J* = 6.5 Hz, 2H), 3.88 (s, 6H), 3.87 (s, 3H), 2.47 (tdd, *J* = 6.3, 4.2, 1.9 Hz, 2H).

^{13}C NMR (101 MHz, $CDCl_3$) δ 169.0, 166.0, 153.5, 145.7, 143.9, 140.1, 130.8, 125.9, 121.2, 105.6, 61.1, 56.3, 41.8, 24.9.

HRMS *m/z* (ESI+) $C_{17}H_{19}NO_5$ ([M+H]+) calculated 317.1263, found 317.1261

1-(3-(3,4,5-trimethoxyphenyl)propanoyl)piperidin-2-one (PL22)

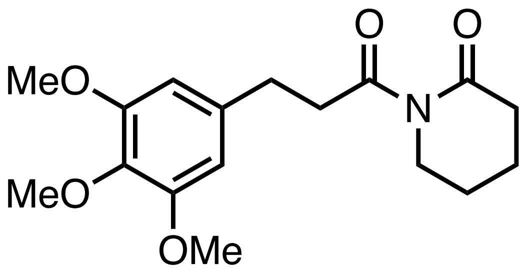

To a solution of (3-(3,4,5-trimethoxyphenyl)propanoic acid (630.7 mg, 2.63 mmol, 1 equiv.), in freshly distilled THF (15 mL) was added triethylamine (0.26 mL, 2.16 mmol, 0.82 equiv.). Pivaloyl chloride (255 μL, 2.07 mmol, 0.79 equiv.) was added at −20 ºC and the reaction mixture was stirred for 45 min. To a separate solution of piperidin-2-one (312.0 mg, 3.15 mmol, 1 equiv.) in freshly distilled THF (15 mL) was added *n*-BuLi (2.4 mL, 3.78 mmol, 1.2 equiv.) at −78 ºC under argon and the reaction was stirred for 45 min. Then, anhydride prepared from the above step was added and the reaction mixture was stirred for 1 h. The reaction mixture was quenched with saturated NH_4Cl (6 mL), extracted with ethyl acetate (2 × 30 mL), the organic layer was separated and washed with sat. NaCl (2 × 18 mL) and dried over anhydrous Na_2SO_4. The residue was evaporated *in vacuo* to give a crude product which was purified by column chromatography on silica gel (3:2 Petrol/EtOAc) to yield **22** as a white powder (524.03 mg, 1.63 mmol, 62%).

R*f* 0.19 (3:2 Petrol/EtOAc).

^{1}H NMR (600 MHz, $CDCl_3$) δ 6.46 (s, 2H), 3.84 (s, 6H), 3.81 (s, 3H), 3.74 – 3.69 (m, 2H), 3.22 (dd, *J* = 8.2, 7.1 Hz, 2H), 2.91 (t, *J* = 7.7 Hz, 2H), 2.56 – 2.51 (m, 2H), 1.82 (ddd, *J* = 8.3, 4.1, 2.0 Hz, 4H).

^{13}C NMR (101 MHz, Acetone) δ 175.31, 172.91, 153.35, 137.21, 136.63, 105.79, 59.51, 55.42, 43.59, 41.10, 34.40, 31.34, 22.15, 20.02.

HRMS *m/z* (ESI+) $C_{17}H_{23}NO_5$ ([M+H]+) calculated 323.1576, found 323.1580

NMR Spectra

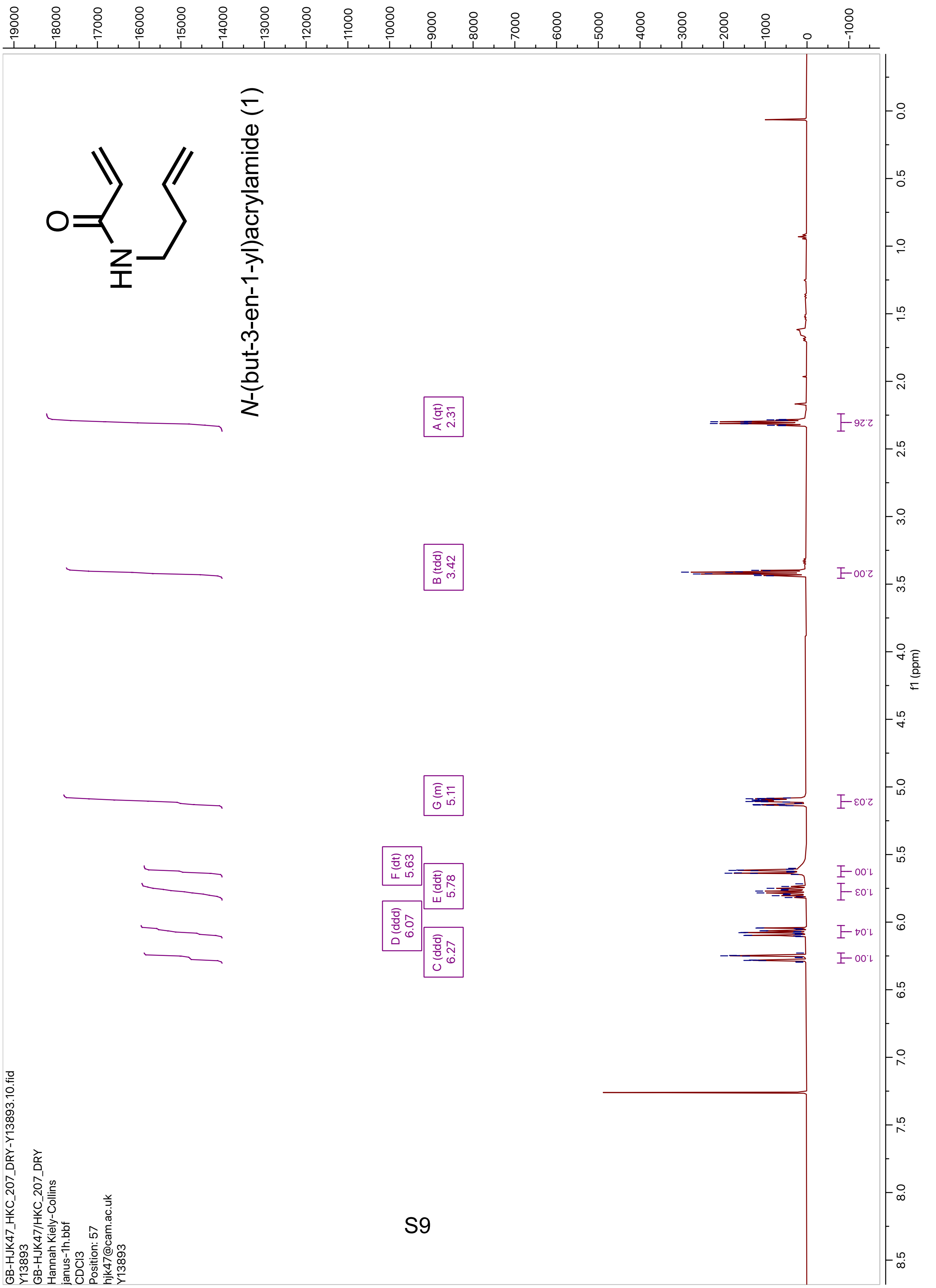

N-(but-3-en-1-yl)acrylamide (1)
HN
O
A (qt)
2.31
B (tdd)
3.42
G (m)
5.11
F (dt)
5.63
E (ddt)
5.78
D (ddd)
6.07
C (ddd)
6.27
2.26
2.00
2.03
1.00
1.03
1.04
1.00
f1 (ppm)
GB-HJK47_HKC_207_DRY-Y13893.10.fid
Y13893
GB-HJK47/HKC_207_DRY
Hannah Kiely-Collins
janus-1h.bbf
CDCl3
Position: 57
hjk47@cam.ac.uk
Y13893

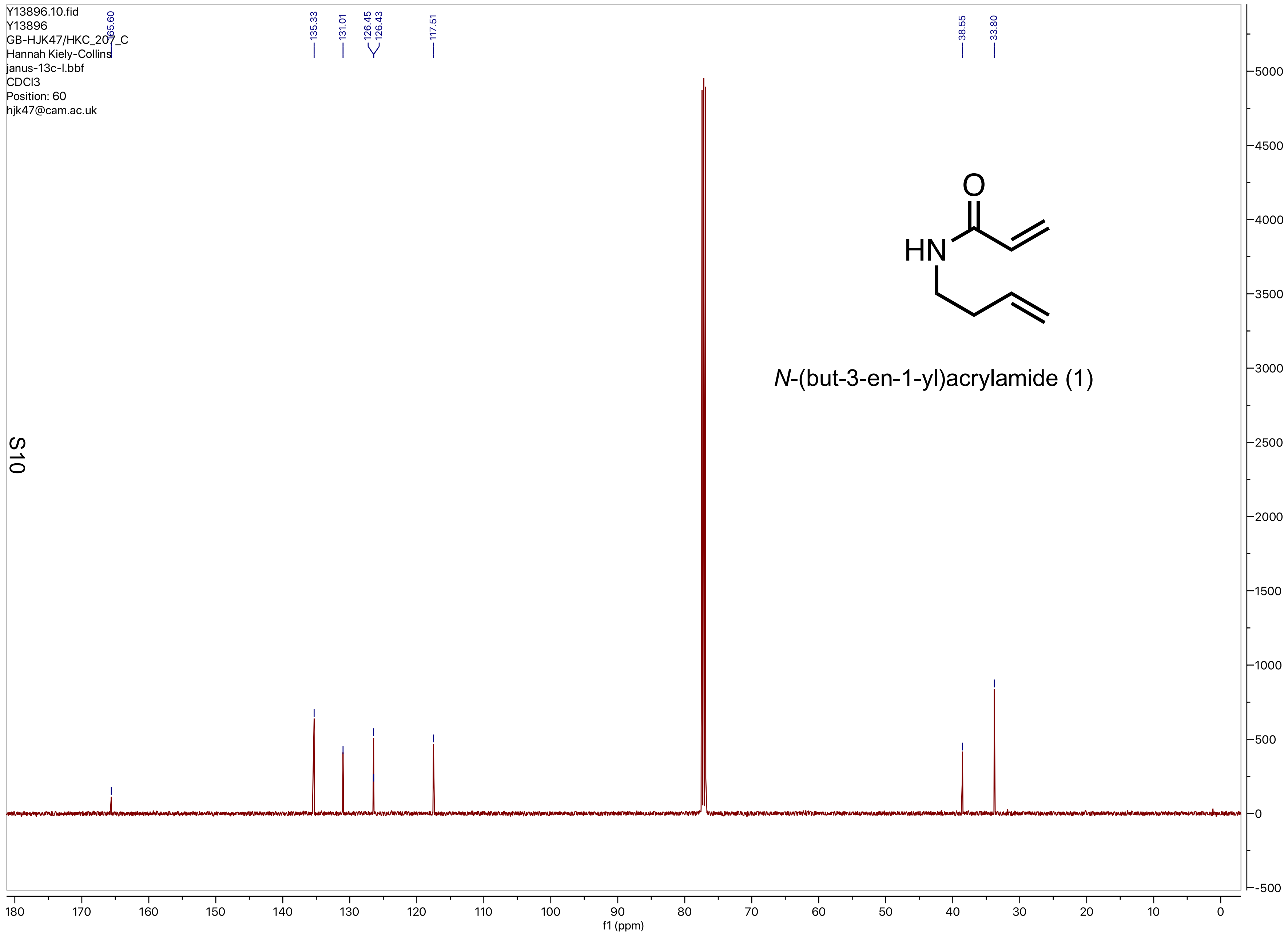
Y13896.10.fid
Y13896
GB-HJK47/HKC_207_C
Hannah Kiely-Collins
janus-13c-l.bbf
CDCl3
Position: 60
hjk47@cam.ac.uk
165.60
135.33
131.01
126.45
126.43
117.51
38.55
33.80
O
HN
N-(but-3-en-1-yl)acrylamide (1)
5000
4500
4000
3500
3000
2500
2000
1500
1000
500
0
-500
180
170
160
150
140
130
120
110
100
90
80
70
60
50
40
30
20
10
0
f1 (ppm)

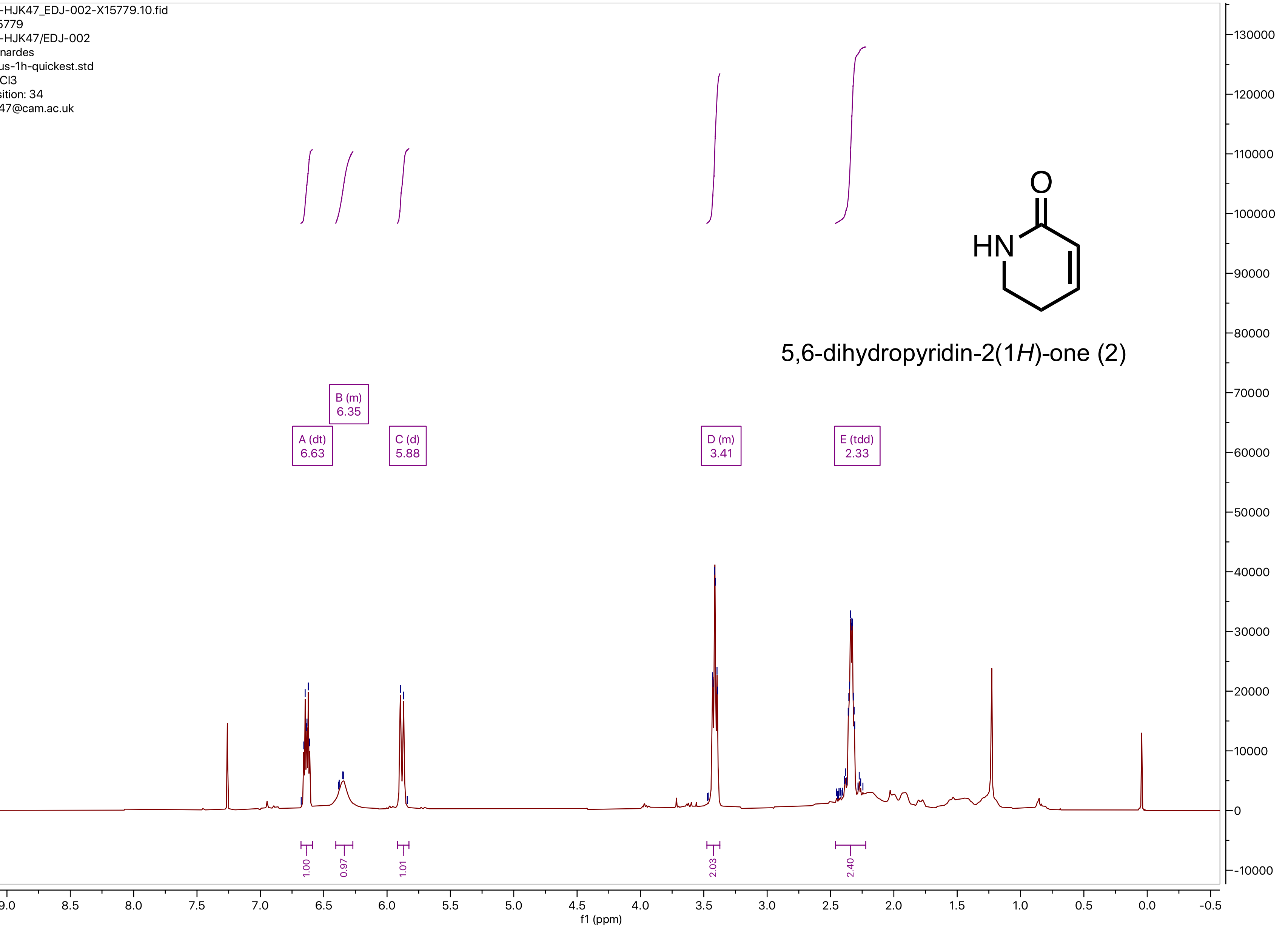
GB-HJK47_EDJ-002-X15779.10.fid
X15779
GB-HJK47/EDJ-002
bernardes
janus-1h-quickest.std
CDCl3
Position: 34
hjk47@cam.ac.uk
HN
O
5,6-dihydropyridin-2(1H)-one (2)
A (dt) 6.63
B (m) 6.35
C (d) 5.88
D (m) 3.41
E (tdd) 2.33
1.00
0.97
1.01
2.03
2.40
f1 (ppm)

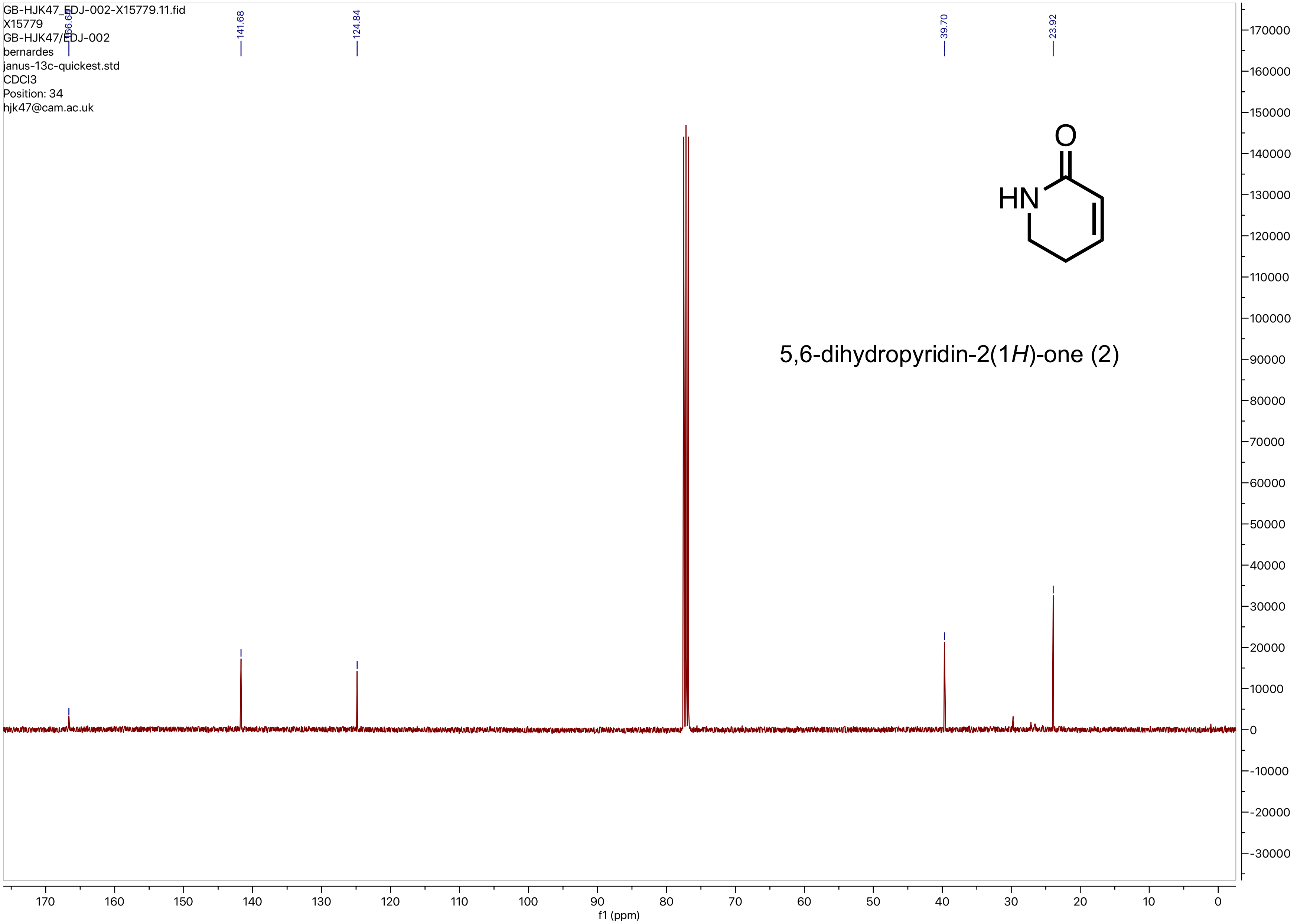
GB-HJK47_EDJ-002-X15779.11.fid
X15779
GB-HJK47/EDJ-002
bernardes
janus-13c-quickest.std
CDCl3
Position: 34
hjk47@cam.ac.uk
141.68
124.84
39.70
23.92
HN
O
5,6-dihydropyridin-2(1H)-one (2)
f1 (ppm)

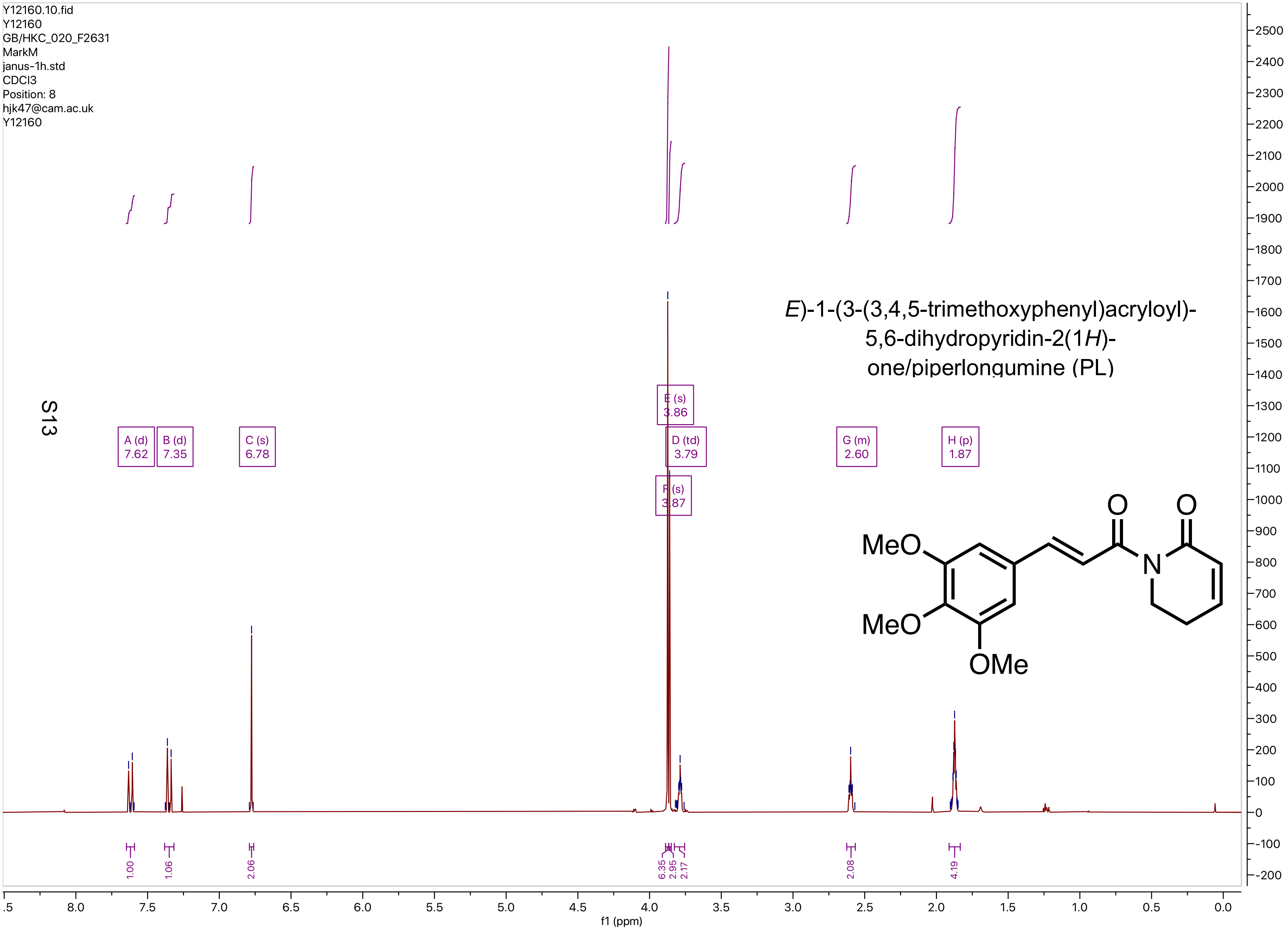
Y12160.10.fid
Y12160
GB/HKC_020_F2631
MarkM
janus-1h.std
CDCl3
Position: 8
hjk47@cam.ac.uk
Y12160
E)-1-(3-(3,4,5-trimethoxyphenyl)acryloyl)-5,6-dihydropyridin-2(1H)-one/piperlongumine (PL)
A (d) 7.62
B (d) 7.35
C (s) 6.78
E (s) 3.86
D (td) 3.79
F (s) 3.87
G (m) 2.60
H (p) 1.87
MeO
MeO
OMe
N
O
O
1.00
1.06
2.06
6.35
2.95
2.17
2.08
4.19
f1 (ppm)

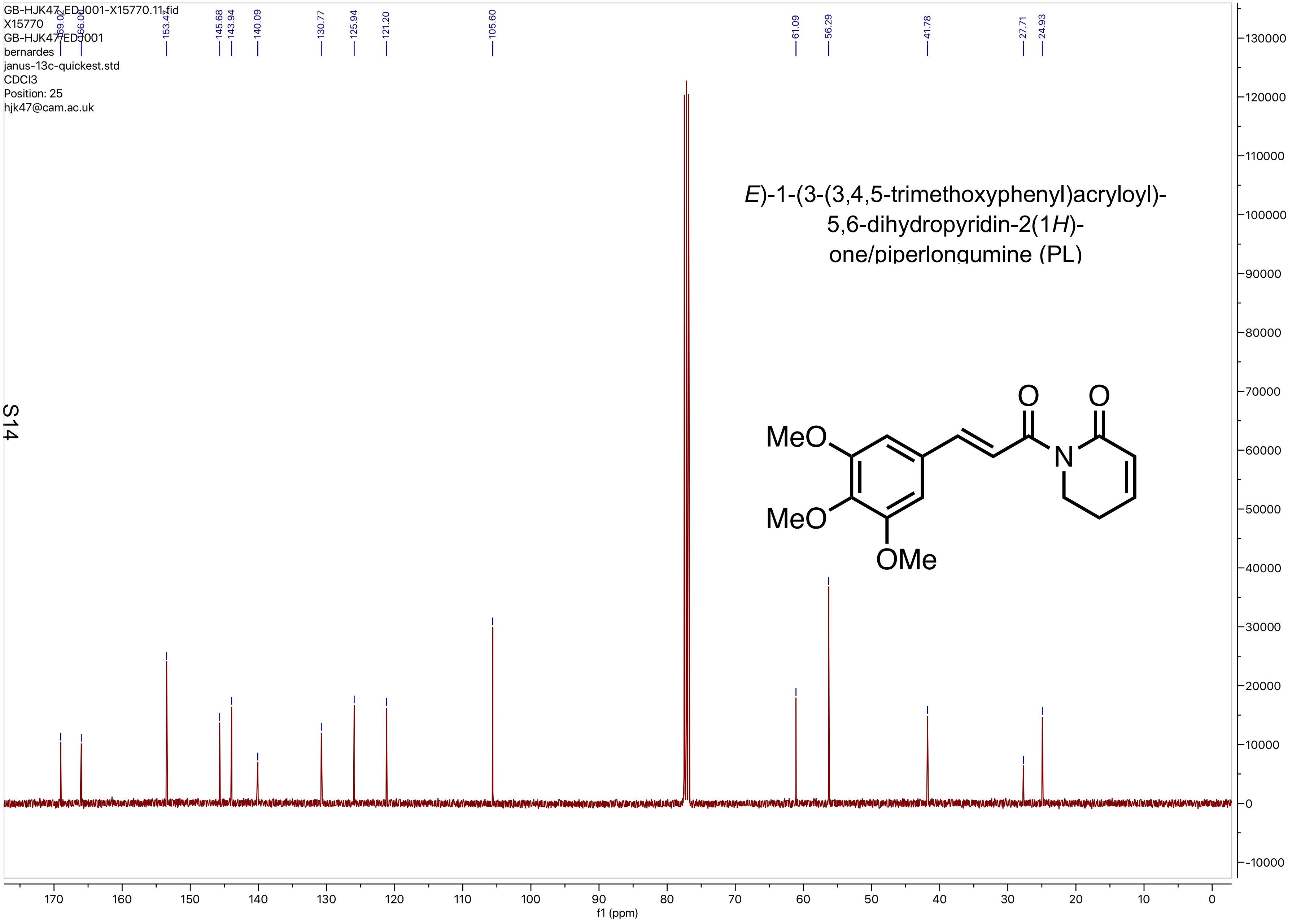
GB-HJK47_EDJ001-X15770.11.fid
X15770
GB-HJK47_EDJ001
bernardes
janus-13c-quickest.std
CDCl3
Position: 25
hjk47@cam.ac.uk
169.02
166.00
153.47
145.68
143.94
140.09
130.77
125.94
121.20
105.60
61.09
56.29
41.78
27.71
24.93
E)-1-(3-(3,4,5-trimethoxyphenyl)acryloyl)-5,6-dihydropyridin-2(1H)-one/piperlongumine (PL)
MeO
MeO
OMe
O
O
N
f1 (ppm)

1-(3-(3,4,5-trimethoxyphenyl)propanoyl)piperidin-2-one (PL22)

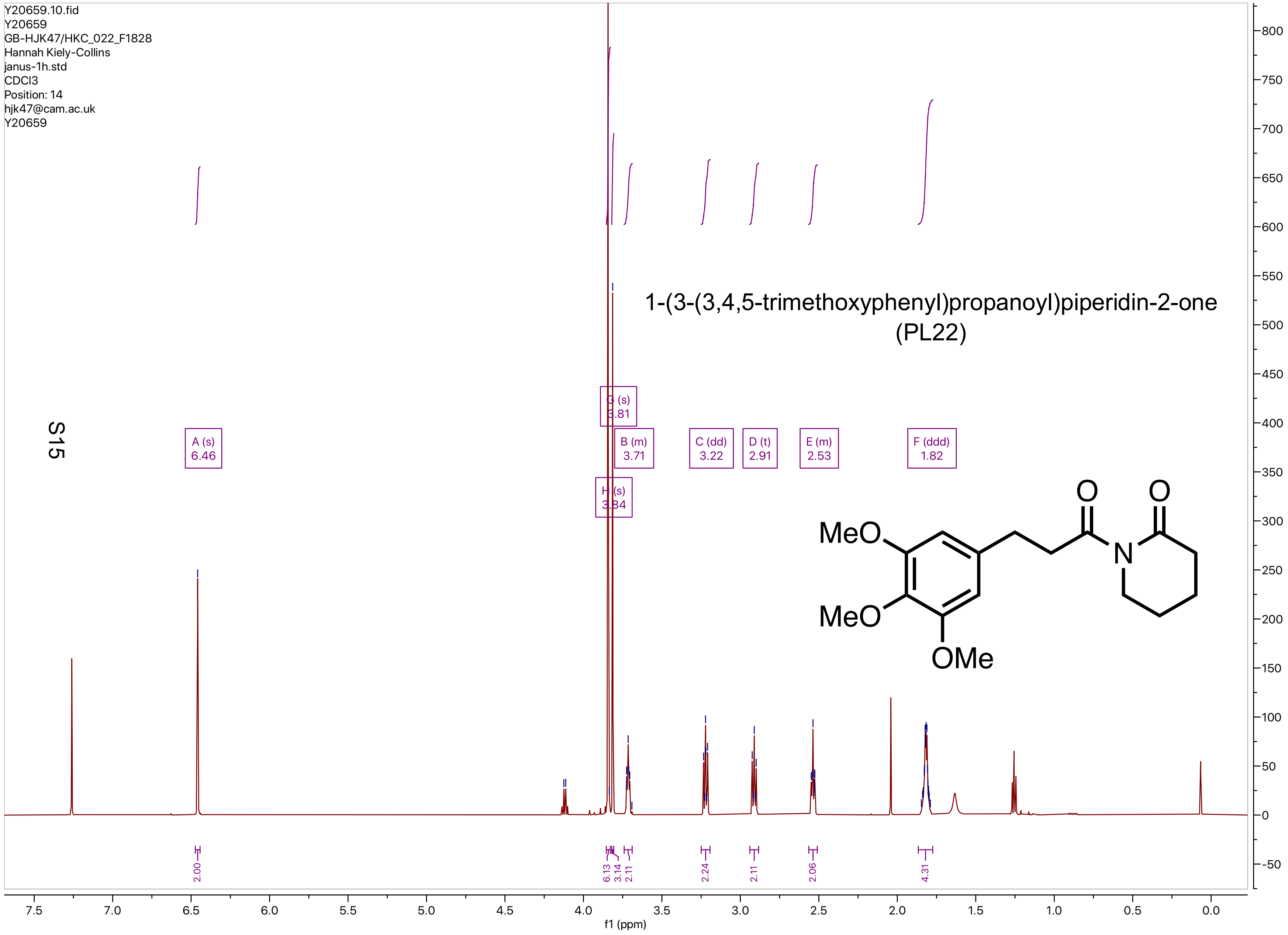

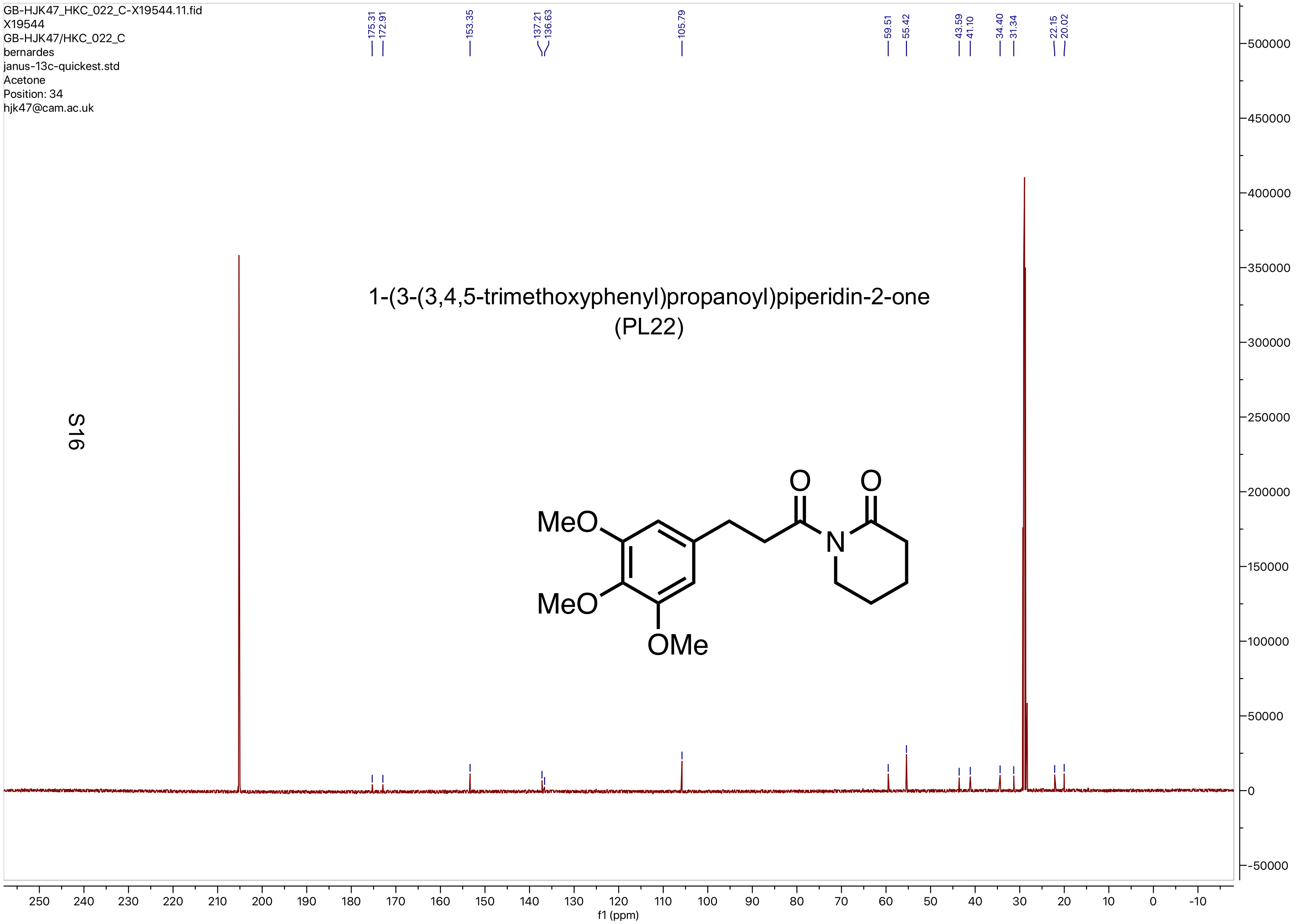

1-(3-(3,4,5-trimethoxyphenyl)propanoyl)piperidin-2-one
(PL22)

REFERENCES:

1. Casey, M.; Leonard, J., LYGO, B. e PROCTER, G. *Advanced Practical Organic Chemistry, Chapman & Hall, New York* **1990**.

2. Meegan, M. J.; Nathwani, S.; Twamley, B.; Zisterer, D. M.; O'Boyle, N. M., Piperlongumine (piplartine) and analogues: Antiproliferative microtubule-destabilising agents. *Eur. J. Med. Chem.* **2017,** *125*, 453–463.
